# Research progress on the role of the locus coeruleus in cognitive function

**DOI:** 10.3389/fnhum.2026.1830622

**Published:** 2026-08-05

**Authors:** Xue Li, Xiaoli Liang, Xinyu Yuan, Ninan Dai, Kun Qian, Ke Li, Yi Zhang, Haiying Wang

**Affiliations:** 1 Department of Anesthesiology, Affiliated Hospital of Zunyi Medical University, Zunyi, Guizhou, China; 2 Department of Anesthesiology, Hospital of Stomatology, Zunyi Medical University, Zunyi, Guizhou, China; 3 School of Anesthesiology, Zunyi Medical University, Zunyi, Guizhou, China; 4 Department of Anesthesiology, The Second Affiliated Hospital of Zunyi Medical University, Zunyi, China; 5 Department of Critical Care Medicine, Affiliated Hospital of Zunyi Medical University, Zunyi, China; 6 Key Laboratory of Anesthesia and Organ Protection of Ministry of Education (In Cultivation), Zunyi Medical University, Zunyi, Guizhou, China; 7 Key Laboratory of Anesthesia and Organ Protection of Guizhou Province, Zunyi Medical University, Zunyi, Guizhou, China; 8 Key Laboratory of Brian Science of Guizhou Provincial Department of Education, Zunyi Medical University, Zunyi, Guizhou, China

**Keywords:** brain network, cognitive function, dopamine (DA), hierarchical regulation, locus coeruleus (LC), noradrenergic (NE)

## Abstract

The locus coeruleus (LC) is the primary source of noradrenergic (NE) neurons in the central nervous system, with extensive neural projections throughout the brain, serving as a key ascending modulatory center for regulating cognitive functions. In recent years, the advances in optogenetics, chemogenetics, and high-resolution neuroimaging techniques—including neuromelanin-sensitive MRI (NM-MRI) and diffusion MRI—have further elucidated the role of the LC in cognitive function regulation. Through the core mechanisms of dynamic tonic–phasic firing mode switching, coordinated co-release of noradrenaline and dopamine (DA), and large-scale brain network reconfiguration, the LC achieves cross-level integrative regulation of fundamental cognitive processes, including learning and memory, vigilance, attention, decision-making, and executive function, with learning and memory processes spanning all levels and providing experiential support for each of these operations. Moreover, the LC is an early vulnerable node for pathological damage in the brain; its structural and functional abnormalities can lead to imbalances in neuromodulator tone, inflammatory-phagocytic imbalance, and disruptions in synaptic plasticity and neurovascular–energy metabolism, thereby driving the onset and progression of pathological cognitive disorders such as Alzheimer’s disease (AD), Parkinson’s disease (PD), dementia with Lewy bodies (DLB), attention-deficit/hyperactivity disorder (ADHD), and autism spectrum disorder (ASD). This article systematically reviews the neurophysiological basis of the LC and its hierarchical regulatory mechanisms over normal cognitive functions, and proposes a unified four-pathway framework through which LC dysfunction gives rise to cognitive impairment across diagnostic boundaries, aiming to deepen the understanding of cognitive neuromodulation theory and provide a theoretical foundation for the early identification and targeted intervention of cognitive disorders.

## 1 Introduction

The locus coeruleus (LC), located in the dorsolateral pontine tegmentum, is the brain region with the highest density of noradrenaline (NE)-producing neurons in the central nervous system (Tsukahara and Engle, 2021). It sends widespread axonal projections to core cognitive brain regions throughout the brain, including the prefrontal cortex (PFC), hippocampus, amygdala, and thalamus (Chen Z. et al., 2023; Huo and Lombardi, 2024; Schwarz et al., 2015; Galgani and Giorgi, 2023). Previous studies defined the LC as an “arousal center” primarily involved in simple physiological processes such as basic vigilance, pain modulation, and stress responses. In recent years, however, the rapid advancement of precise neuromodulation techniques such as optogenetics and chemogenetics, together with high-resolution imaging techniques—most notably high-resolution neuromelanin-sensitive magnetic resonance imaging (NM-MRI)—has greatly expanded our understanding of LC’s function. Neuromelanin is an endogenous pigment that accumulates in catecholaminergic neurons, including LC noradrenergic neurons, as a byproduct of iron-dependent catecholamine oxidation (Trujillo et al., 2024; Ericson, 2026). Normal LC neurons stably accumulate this pigment. NM-MRI [also referred to as LC-sensitive MRI (Dahl et al., 2022)] exploits its paramagnetic properties to generate T1-weighted hyperintense contrast, thereby enabling non-invasive *in vivo* assessment of LC neuronal integrity and long-term noradrenergic system activity. Reduced signal intensity reflects LC neuronal degeneration, neuromelanin depletion, and compromised LC structural integrity (Ericson, 2026; Dahl et al., 2022). In parallel, high spatial and angular resolution diffusion MRI has enabled the reconstruction of LC-originating fiber tracts and the *in vivo* assessment of LC structural connectivity, offering new opportunities to link LC microstructural integrity with cognitive performance (Valdés Cabrera et al., 2026; Solders et al., 2022). High-field functional MRI, positron emission tomography (PET), and electrophysiological approaches have further expanded the multimodal toolkit for probing LC functional dynamics and its role in cognition (Sun et al., 2020). These advances have revealed that the LC also participates in the regulation of multi-dimensional higher-order cognitive processes—including learning and memory, attention, decision-making, and executive function—through multi-transmitter release, dynamic modulation of firing patterns, and brain network reconfiguration, thereby establishing it as a key neuromodulatory hub for cognitive processing in the brain.

Cognitive function serves as a critical foundation for individuals to achieve environmental adaptation, goal-directed behavior, and social functioning, encompassing five core dimensions: learning and memory, vigilance, attention, decision-making, and executive function (Mahoney and Schmidt, 2024). These dimensions do not operate in isolation; rather, they form a progressively layered and mutually coordinated whole. Vigilance provides the fundamental arousal substrate for all cognitive processing, attention enables selective processing of environmental information, decision-making accomplishes the strategic selection of filtered information, and executive function translates decision strategies into concrete behavior. Learning and memory processes permeate the entire sequence, providing experiential reference for the dynamic adjustment of each cognitive stage. Existing research has largely focused on the critical role of the LC in the regulation of individual cognitive functions; however, a systematic theoretical framework for how it achieves hierarchical integrated regulation across multiple cognitive domains has yet to be established. Moreover, the LC is recognized as an early vulnerable node for pathological damage in the brain. At the pathological level, growing evidence indicates that the LC is among the earliest affected brain regions in several neurological disorders. Significant LC neuronal loss and noradrenergic dysfunction have been reported in Alzheimer’s disease (AD) (Ciampa et al., 2022), Parkinson’s disease (PD) (Sun et al., 2025), and dementia with Lewy bodies (DLB) (Železníková et al., 2025), with such alterations closely associated with declines in memory, attention, and executive function (Ciampa et al., 2022; Sun et al., 2025; Železníková et al., 2025). In addition, dysfunction of the LC–NE system has been closely associated with cognitive impairments observed in neurodevelopmental disorders, including attention-deficit/hyperactivity disorder (ADHD) and autism spectrum disorder (ASD) (Hinshaw, 2018). Despite these converging findings, the link between disruption of its normal regulatory mechanisms and pathological cognitive disorders still lacks a systematic integration that spans from “physiological regulation” to “pathological imbalance.”

Based on this, the present article proceeds from the neurophysiological basis of the LC to systematically dissect its hierarchical regulatory mechanisms over learning and memory, vigilance, attention, decision-making, and executive function, delineate the progressive and synergistic relationships among these cognitive functions, and concurrently outline the common pathological pathways through which LC dysfunction gives rise to cognitive disorders, with the aim of constructing a comprehensive theoretical framework for LC-mediated cognitive regulation and providing a novel perspective for basic research in cognitive neuroscience and clinical intervention for cognitive disorders.

## 2 Neurophysiological basis of the locus coeruleus

As a key ascending modulatory nucleus in the central nervous system, the LC’s distinct anatomical projection characteristics, firing patterns, capacity for multi-transmitter release, and brain network regulatory capability collectively constitute the neurophysiological foundation for the regulation of cognitive functions and serve as the core prerequisite for its hierarchical modulation of cognition.

### 2.1 Anatomical structure and widespread projection characteristics

The noradrenergic neurons in the central nervous system originate primarily from the LC (Tsukahara and Engle, 2021). The LC is not a homogeneous structure. Cytoarchitectonic studies by Swanson (1976) have subdivided the nucleus into a dorsal division, where neurons are densely packed and predominantly fusiform in shape, and a ventral division, characterized by larger multipolar neurons. Although the LC contains only approximately 1,600 neurons (with the ventral division contributing merely about 210 of these), it gives rise to remarkably widespread projections, spanning the hippocampus, prefrontal cortex, amygdala, thalamus, cerebellum, and spinal cord (Chen Z. et al., 2023; Huo and Lombardi, 2024; Schwarz et al., 2015; Galgani and Giorgi, 2023). This “sparse neurons–widespread projections” architecture enables the LC to broadly influence neural activity throughout the brain. Notably, despite being viewed as a globally projecting system, retrograde tracing studies have revealed a fine topographic organization: neurons in the dorsal division project predominantly to cognition-related regions such as the hippocampus and prefrontal cortex, whereas those in the ventral division mainly target the cerebellum and spinal cord (Loughlin et al., 1986). This spatial compartmentalization provides a structural basis for understanding the differential modulatory functions of LC subregions. Furthermore, LC–NE fibers are predominantly distributed in a diffuse pattern within the cortex and release NE via volume transmission from non-classical synapses (Toyoda et al., 2022), thereby transcending the spatial constraints of classical synaptic transmission. This mode of signaling enables synchronous modulation of neuronal population excitability and the signal-to-noise ratio (SNR), providing the anatomical foundation for cognitive processes that require rapid whole-brain coordination, such as attentional orienting and the maintenance of arousal. While the anatomical organization of LC projections has been classically characterized by post-mortem tracing and immunohistochemistry, these methods are inherently limited to ex vivo specimens and cannot capture individual variability *in vivo*. Recent advances in high spatial and angular resolution diffusion MRI have made it possible to reconstruct LC-originating fiber tracts and assess LC structural connectivity in the living human brain, offering new opportunities to link LC microstructural integrity with cognitive performance (Valdés Cabrera et al., 2026; Solders et al., 2022; Sun et al., 2020). Nevertheless, the small size and deep pontine location of the LC continue to pose significant challenges for *in vivo* imaging, necessitating specialized high-resolution acquisition and analysis strategies.

### 2.2 Dynamic switching between tonic and phasic firing modes

LC neuronal activity is primarily characterized by two modes—tonic firing and phasic firing—which serve distinct functional roles under different cognitive states. The dynamic switching between tonic and phasic firing constitutes a key mechanism through which the LC regulates cognitive functions. Tonic firing is closely associated with the baseline arousal level and overall vigilance state of the organism (Kim et al., 2026; James et al., 2021). Moderate tonic firing levels help maintain stable attention and task engagement; excessively low levels tend to lead to attentional lapses and sluggish responses, whereas excessively high levels can trigger anxiety and reduced cognitive flexibility (Unsworth and Robison, 2017); Phasic firing manifests as brief, high-frequency burst discharges that typically occur in response to novel stimuli, reward prediction errors, or task-critical events (Kim et al., 2026; James et al., 2021; Sales and Friston, 2019), and can transiently enhance the neural representation strength of target-related information, thereby facilitating attentional focusing (James et al., 2021; Babushkina and Manahan-Vaughan, 2025), memory encoding (James et al., 2021), and rapid decision-making (Aston-Jones and Cohen, 2005). Aston-Jones and Cohen’s “adaptive gain theory” further elucidates the regulatory relationship between these two modes: through dynamic switching between tonic and phasic firing, the LC helps individuals strike a balance between “exploration” and “exploitation” strategies (Aston-Jones and Cohen, 2005), offering a perspective for understanding the central role of the LC in cognitive processes such as attention and decision-making.

### 2.3 Neuromodulatory mechanisms of noradrenaline

Noradrenaline released from the LC acts on α1-, α2-, and *β*-adrenergic receptors on neurons in target brain regions, influencing neuronal excitability and synaptic transmission through multiple mechanisms (Zhou and Chu, 2025). In the cortex and hippocampus, NE enhances the amplitude of postsynaptic responses by activating β-adrenergic receptors while simultaneously suppressing background noise firing in neurons, thereby increasing the SNR ratio of sensory inputs and making task-relevant information more prominent within neural networks. Furthermore, NE can modulate neuronal gain—altering the slope of neuronal responses to input stimuli—and thus influence the efficiency of information integration. This modulation exhibits a characteristic inverted U-shaped profile: moderate NE levels help maintain the stability and flexibility of neural networks, whereas either excessive or insufficient levels disrupt this balance and lead to cognitive decline. This inverted U-shaped effect has been observed across various cognitive tasks, including attention and working memory (Aston-Jones and Cohen, 2005). In terms of synaptic plasticity, NE participates in the regulation of long-term potentiation (LTP) and long-term depression (LTD) through activation of *β*-adrenergic receptors, providing a molecular foundation for the formation and stabilization of learning and memory (James et al., 2021; Babushkina and Manahan-Vaughan, 2022).

### 2.4 Dopamine co-release and its functional significance

Conventional views hold that central DA is primarily released by the ventral tegmental area (VTA) and substantia nigra pars compacta (SNc). However, recent studies have revealed that, under specific physiological conditions, LC neurons can co-release DA alongside NE, a phenomenon particularly prominent in LC projection pathways to the hippocampus and PFC (Devoto et al., 2005). LC-derived DA activates D1/D5 receptors on target neurons, enhancing the activity of the cAMP–PKA–CREB signaling pathway, thereby promoting synaptic plasticity and memory formation. It plays a more direct regulatory role in stabilizing LTP and facilitating long-term memory consolidation (Takeuchi et al., 2016; Lynch, 2004; Morris, 2003; Deng et al., 2025; Kempadoo et al., 2016). The coordinated release of NE and DA endows the LC with both rapid modulation of brain states (mediated by NE) and long-term regulation of neuroplasticity (mediated by DA) during cognitive processing, further enriching its neuromodulatory repertoire and serving as a key nexus linking short-term cognitive regulation to long-term learning and memory.

### 2.5 Regulation of large-scale brain networks

The LC not only modulates single neurons or local neural circuits but also contributes to the regulation of overall cognitive states by modulating functional coupling among different brain networks. Functional neuroimaging studies have shown that when LC activity increases, functional connectivity within the executive control network (ECN) and the salience network (SN) is significantly enhanced, whereas activity in the default mode network (DMN) is suppressed (Geng et al., 2024). This brain network reconfiguration facilitates a shift from an introspective/mind-wandering resting state to a task-oriented/goal-focused cognitively engaged state. Specifically, the SN is responsible for detecting salient or unexpected stimuli in the environment and triggering the reallocation of attentional resources. By enhancing the responsiveness of this network, the LC enables individuals to react more rapidly to critical events, providing network-level support for the optimal allocation of whole-brain cognitive resources (Geng et al., 2024; Rodenkirch et al., 2019).

**Figure 1:**
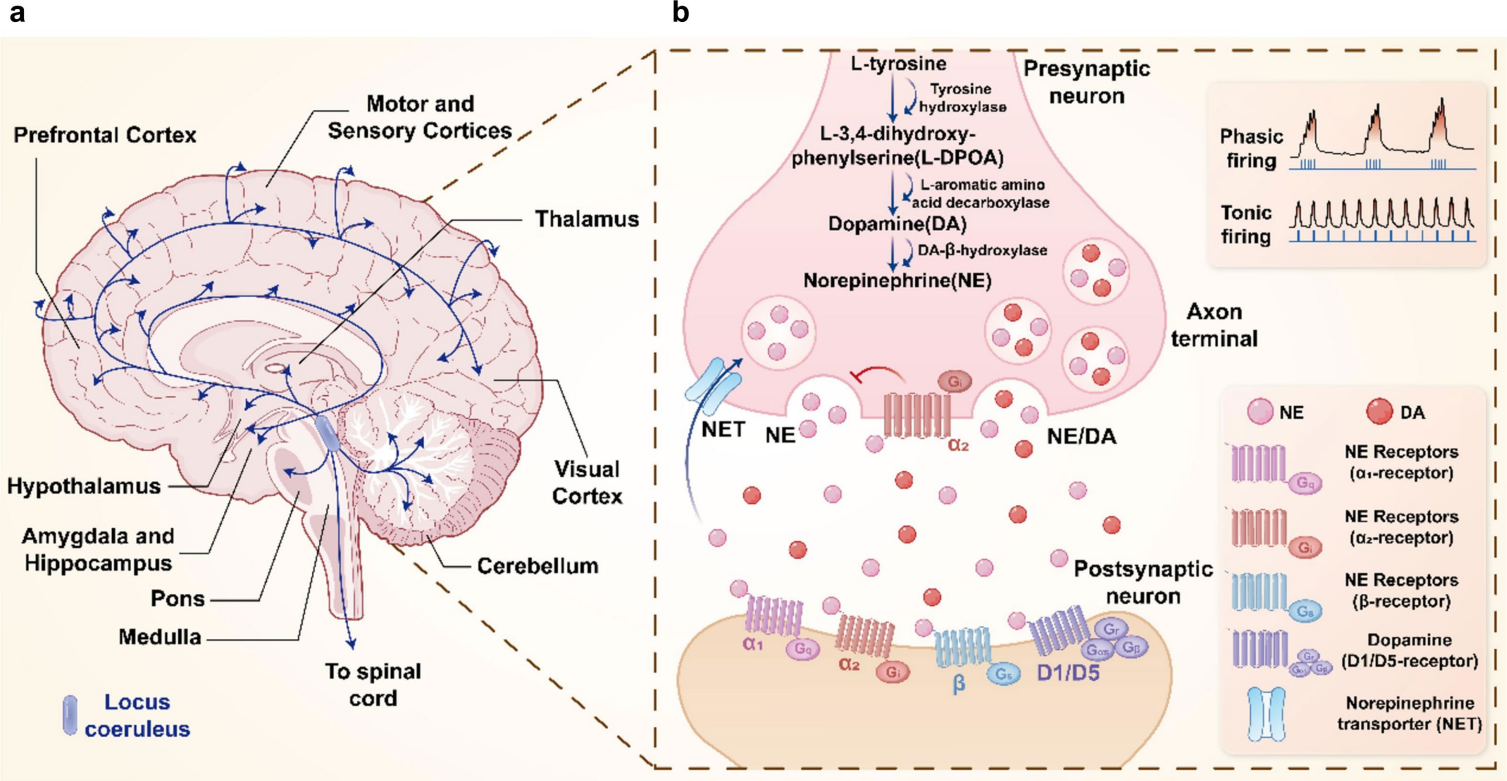
Structural organization and cellular neuromodulatory mechanisms of the LC. This figure illustrates the anatomical distribution, firing dynamics, and synaptic neuromodulatory mechanisms of the LC. Panel **(a)** shows the anatomical location of the LC in the dorsolateral pontine tegmentum and its extensive ascending and descending projections to widespread cortical and subcortical targets, including the PFC, thalamus, hippocampus, amygdala, cerebellum, motor and sensory cortices, hypothalamus, and spinal cord. This “limited neurons–broad projections” architecture enables the LC to function as a global modulatory hub coordinating large-scale brain states. Panel **(b)** depicts neurotransmitter synthesis, release, and receptor-mediated signaling at LC axon terminals. NE is synthesized from L-tyrosine via sequential enzymatic conversion, with DA serving as an intermediate and, under specific physiological conditions, a co-released transmitter. Released catecholamines act on postsynaptic adrenergic (α1, α2, β) and dopaminergic (D1/D5) receptors to regulate neuronal excitability, synaptic plasticity, and neural gain. NE signaling is terminated through reuptake by the NET. LC neurons exhibit tonic and phasic firing modes, which dynamically regulate arousal state, signal salience, and information processing efficiency. Together, these anatomical and molecular properties provide the neurophysiological substrate for LC-mediated large-scale modulation of brain function and cognitive state regulation. Abbreviations used in this figure: all terms are defined in earlier sections of the main text.

In summary, the LC’s widespread projections throughout the brain provide the anatomical foundation for its global regulatory capacity. The dynamic switching between tonic and phasic firing modes serves as a key mechanism for modulating cognitive states. The coordinated co-release of NE and DA enables the LC to accomplish both short-term state modulation and influence long-term plasticity. Moreover, the LC participates in large-scale brain network reconfiguration, thereby integrating local neural activity into overall cognitive states. Together, firing mode regulation, coordinated multi-transmitter modulation, and brain network reconfiguration constitute the core mechanisms through which the LC regulates cognitive functions, laying the neurophysiological foundation for its hierarchical modulation of cognition (Figure 1).

## 3 Locus coeruleus and learning and memory

Learning and memory constitute a core component of cognitive function, encompassing three stages: encoding, consolidation, and retrieval. They not only serve as the foundation for individuals to accumulate experience and shape adaptive behavior but also permeate other cognitive processes such as vigilance, attention, decision-making, and executive function. Through dynamic switching of firing modes (Babushkina and Manahan-Vaughan, 2022), coordinated co-release of NE and DA, and coupling across brain-wide networks (Takeuchi et al., 2016; Sara, 2009; Mather et al., 2016), the LC systematically contributes to the entire process of learning and memory, which in turn represents an essential prerequisite for the LC to support other higher-order cognitive functions.

### 3.1 Memory encoding

Memory encoding is the process of transforming external information into neural representations in the brain, and its efficiency is primarily influenced by arousal levels, the allocation of attentional resources, and contextual salience. The LC regulates these factors through its extensive projection network, thereby determining which information is preferentially encoded and retained over the long term (Clewett et al., 2025; Langley and Hussain, 2022; Luskin and Li, 2025). When novel stimuli, rewards, or task-relevant events occur, the LC generates phasic firing, transiently increasing NE/DA release. On the one hand, this enhances the strength of neural representations of target-related information in the cortex and hippocampus, improves the signal-to-noise ratio, and allows important information to be prioritized (James et al., 2021; Aston-Jones and Cohen, 2005; Servan-Schreiber et al., 1990). On the other hand, NE enhances hippocampal neuronal excitability by activating *β*-adrenergic receptors, thereby increasing the probability of LTP induction (O'dell et al., 2015); meanwhile, DA, through activation of D1/D5 receptors, initiates the cAMP–PKA–CREB signaling pathway, providing the synaptic plasticity foundation for the initial neural representation of memory (Otmakhova and Lisman, 1998; Rivera-Maya et al., 2024). Furthermore, the LC’s regulation of synaptic plasticity is frequency-dependent: low-frequency stimulation tends to induce LTD, whereas high-frequency stimulation promotes LTP formation (Babushkina and Manahan-Vaughan, 2022), enabling the LC to dynamically adjust the priority of information encoding based on contextual salience. In addition, the LC is involved in detecting “event boundaries”: when significant changes occur in the external context, its phasic activity increases, segmenting continuous experience into discrete memory units that are more readily stored, thereby enhancing the organization and retrieval efficiency of episodic memory (Clewett et al., 2025) Moreover, NE released from the LC can activate α1A-adrenergic receptors on astrocytes, and through glial-mediated metabolic and signaling modulation, further regulate synaptic strength, enriching the regulatory mechanisms of memory encoding (Lefton and Wu, 2025).

### 3.2 Memory consolidation

Newly formed memory traces are labile and must undergo consolidation before they can be transformed into stable, long-term neural representations. This process requires the maintenance of synaptic plasticity, the synthesis of plasticity-related proteins (PRPs), and cross-regional brain network reorganization. LC activity, which is associated with stimulus novelty and behavioral relevance, serves as a key modulatory hub for memory consolidation. NE and DA released by the LC act through *β*-adrenergic and D1/D5 receptors to initiate and amplify the cAMP–PKA–CREB signaling cascade, promoting the synthesis and distribution of PRPs. At the molecular level, this reinforces the synaptic potentiation established during the encoding phase, achieves long-term stabilization of synaptic plasticity, and provides the molecular basis for the conversion of transient memory into long-term memory (O'dell et al., 2015; Otmakhova and Lisman, 1998; Rivera-Maya et al., 2024). Within the “synaptic tagging and capture” framework, LC signaling expands the pool of available PRPs, making it easier for activated synapses to “capture” these proteins and sustain long-term synaptic potentiation (Moncada, 2017)—a mechanism that explains why salient or emotionally charged experiences are more readily consolidated into long-term memory. At the network level, the LC modulates functional coupling within the hippocampal–cortical network to facilitate memory consolidation: during wakefulness, phasic LC activity enhances the efficiency of information transfer between the hippocampus and the PFC, laying the groundwork for consolidation (Kempadoo et al., 2016); during slow-wave sleep, LC activity is markedly reduced, which favors the coordination between hippocampal sharp-wave ripples (SWRs) and cortical spindles, thereby promoting the transfer of memory traces to the neocortex. Artificially enhancing LC activity during this period disrupts interregional coupling and impairs memory consolidation (Novitskaya et al., 2016). Moreover, LC–NE signaling regulates astrocytic lactate metabolism, providing energetic support for long-term synaptic plasticity and promoting memory stabilization (Gao et al., 2016).

### 3.3 Memory retrieval

Memory retrieval is the process of reactivating stored information and applying it to ongoing behavior, and its efficiency depends on the excitability state of the relevant neural circuits. Regulation of memory retrieval by the LC–NE system exhibits an inverted U-shaped characteristic: moderate activation of the LC enhances the excitability of hippocampal and amygdala neurons through α1- and *β*-adrenergic receptors, thereby increasing the probability of successfully reinstating memory cues; however, excessively high or low NE release disrupts the retrieval process, leading to reduced retrieval efficiency or diminished accuracy (Fukabori and Iguchi, 2020; Arnsten, 2011). In spatial and contextual memory, the LC–hippocampal pathway plays a critical role. Optogenetic activation of LC projections to hippocampal CA1 has been shown to enhance LTP in this region and restore memory retrieval ability in animal models of cognitive impairment (Gálvez-Márquez et al., 2025). Moreover, the LC can enhance the pattern separation capacity of the dentate gyrus, thereby improving the discriminability of similar contextual cues and increasing the precision of memory retrieval (Clewett et al., 2025). The PFC, in turn, influences neuronal activity in the hippocampus and amygdala through top-down regulation and participates in strategy selection during memory retrieval (Anderson et al., 2016), while NE released from LC inputs to the PFC can enhance error monitoring and behavioral adjustment abilities in this region, optimizing the contextual adaptability of retrieval and enabling individuals to flexibly adjust memory retrieval strategies according to the current environment (Matchett and Grinberg, 2021).

In summary, the LC regulates the processes of memory encoding, consolidation, and retrieval through transient phasic firing modulation, coordinated NE/DA signaling, and cross-regional brain network coupling. Among these, the dynamic balance between tonic and phasic firing modes lies at the core of LC-mediated memory regulation. The release of NE and DA is both frequency-dependent and state-dependent, determining which memories are selectively retained over the long term and shaping the stability of memory traces. These mechanisms provide the foundation for the implementation of higher-order cognitive functions such as vigilance and attention.

## 4 Locus coeruleus and alertness

Alertness is the core capacity to maintain wakefulness, respond rapidly to external stimuli, and prepare the brain for information processing, serving as the physiological foundation for all higher-order cognitive functions, including learning and memory, attention, and decision-making (Oken et al., 2006). Without a stable vigilant state, subsequent cognitive processes such as information selection and strategy formulation cannot proceed efficiently. The LC and its noradrenergic system constitute a key neuromodulatory hub involved in vigilance regulation in the brain (Liebe et al., 2022). Through multi-level mechanisms—including firing mode switching, brain network modulation, and noradrenaline release—the LC dynamically shapes the organism’s vigilant state, enabling a flexible transition between the maintenance of baseline arousal and rapid responses to unexpected stimuli.

### 4.1 Neurodynamic foundations of firing patterns and alertness states

The tonic–phasic dual firing modes of LC neurons constitute the core neurodynamic basis for the regulation of vigilance states, and their synergistic interaction enables fine-tuned modulation of vigilance levels. Tonic firing reflects the individual’s overall arousal level and baseline vigilance state, and its firing frequency is positively correlated with baseline vigilance; however, this relationship also follows an inverted U-shaped pattern: moderate tonic firing helps maintain stable arousal and sustained attention, placing the individual in an optimal “ready-to-respond” state; when tonic firing is too low, individuals are prone to drowsiness, attentional lapses, and sluggish responses, reflecting insufficient baseline vigilance; when it is excessively high, it triggers hyperarousal, anxiety, and reduced cognitive efficiency, leading to a state of “high vigilance but low efficiency” (Unsworth and Robison, 2017; Sales and Friston, 2019). Phasic firing, in contrast, manifests as brief, high-frequency bursts, typically triggered by novel stimuli, unexpected events, or behaviorally significant cues. It can transiently enhance the signal-to-noise ratio of sensory systems, enabling the individual to rapidly switch from a baseline vigilant state to a heightened state of alertness when the environment changes, quickly focus on critical stimuli, and achieve efficient responses to unexpected information (Unsworth and Robison, 2017; Sales and Friston, 2019). This dual tonic–phasic regulatory mechanism allows the LC to flexibly switch between “maintaining baseline vigilance” and “responding to unexpected stimuli,” providing the physiological foundation for stable and efficient cognitive processing.

### 4.2 Central role in the attention and alertness network

From a brain network perspective, the regulation of vigilance primarily depends on the vigilance network composed of the thalamus, PFC, and parietal cortex, and the LC–NE system serves as an important modulatory node within this network by influencing the excitability and functional coupling of these brain regions (Torres et al., 2025). During quiet wakefulness, the LC maintains low-frequency tonic firing and, through NE release, modulates the neuronal excitability of the thalamus, PFC, and parietal cortex, keeping the brain in a background “ready-to-respond” state; at this stage, the vigilance network is maintained at a low level of activation, which ensures sensitivity to external stimuli while preventing excessive energy consumption. When novel or salient stimuli appear, the LC rapidly generates phasic firing, leading to a transient surge in NE release that markedly enhances the information transmission efficiency of thalamocortical pathways, increases the individual’s sensitivity and response speed to external stimuli, and concurrently promotes high-intensity activation of the vigilance network, swiftly shifting the individual into a state of heightened alertness (Aston-Jones and Cohen, 2005). Functional neuroimaging studies have revealed that when LC activity is enhanced, functional connectivity between the thalamus and PFC is significantly strengthened, whereas activity in the DMN is suppressed—a network reconfiguration that facilitates a rapid shift from an internally directed default mode state to an externally task-oriented vigilant state, enabling the swift reallocation of cognitive resources to external stimuli (Geng et al., 2024) Furthermore, phasic activation of the LC can attenuate the acoustic startle response (ASR), indicating its important role in sensorimotor gating and rapid defensive reactions, and further corroborating its central role in the regulation of vigilance states.

### 4.3 Hierarchical mechanisms underlying the modulation of alertness

Through the release of NE, the LC achieves comprehensive regulation of the organism’s vigilance state across three levels: sensory processing, cognitive control, and energy regulation. At the sensory processing level, NE enhances the signal-to-noise ratio of sensory cortical neurons, enabling threat- or reward-related stimuli to receive prioritized processing resources and thereby improving the individual’s ability to identify critical stimuli (Mather et al., 2016). At the cognitive control level, NE strengthens functional connectivity between the PFC and thalamus, reinforcing top-down cognitive regulation, supporting sustained attention and rapid response preparation, and enabling the individual to maintain goal-directed behavior under high-vigilance conditions (Torres et al., 2025). At the energy regulation level, the LC modulates blood flow supply to key brain regions by influencing the neurovascular coupling mechanism, thereby providing adequate metabolic and energetic support for cognitive processing during heightened vigilance and preventing a rapid decline in vigilance due to energy insufficiency (Sun et al., 2026). Simultaneously, the LC dynamically adjusts NE release levels according to task demands, enabling temporal regulation of vigilance states: during tasks with high cognitive load or high vigilance requirements, LC NE release increases, rapidly shifting attention from a relatively “sluggish” state to a state of “heightened alertness”; during low-vigilance demands or rest, LC activity declines and NE release decreases, facilitating the brain’s entry into a low-energy-consumption mode and providing conditions for sleep and physiological recovery (Aston-Jones and Bloom, 1981). This “on-demand” regulatory characteristic enables the LC to strike a balance between cognitive efficiency and energy expenditure, serving as a crucial safeguard for the organism’s adaptation to complex environments.

In summary, through the dynamic switching between tonic and phasic firing modes, the LC achieves fine-tuned regulation of vigilance levels; by modulating the functional coupling of the vigilance network, it accomplishes the transition from the brain network level to overall cognitive states; and through multi-level NE release, it shapes the stability, flexibility, and energy efficiency of the vigilant state. The stable vigilance state mediated by the LC lays the foundation for subsequent selection, maintenance, and shifting of attention, representing a critical link in the cognitive progression from “basic arousal” to “information selection.”

## 5 Locus coeruleus and attention

Attention is the core capacity to select, sustain, and flexibly shift cognitive resources among multiple internal and external stimuli, serving as a critical hub linking basic arousal (vigilance) to higher-order cognitive decision-making (Burgoyne and Engle, 2020). Building upon a stable vigilant state, attention enables selective information processing that filters out key information for subsequent decision-making and executive functions, thereby avoiding wasteful expenditure of cognitive resources. The traditional view holds that attention is primarily regulated by a “top-down” control network composed of the PFC and parietal cortex. However, recent studies have confirmed that the LC, through its characteristic firing patterns, neurotransmitter release, and brain network reconfiguration mechanisms, plays a critical modulatory role in the dynamic regulation of attention, enabling a functional transition from basic vigilance to oriented attention, and serving as a key regulatory target for the modulation of vigilance levels, prioritization of salient stimuli, and allocation of attentional resources.

### 5.1 Firing patterns and attentional state switching

The dual tonic–phasic firing modes of LC neurons constitute the core mechanism for the dynamic regulation of attentional states, and their dynamic switching determines both the stability and flexibility of attention, in close alignment with the “adaptive gain theory”: tonic firing levels reflect the overall arousal state and degree of task engagement, with moderate tonic activity helping to sustain attention and enabling prolonged focus on the current task; phasic firing, by contrast, typically increases transiently upon the appearance of novel stimuli or task-critical events, facilitating rapid focusing on target-related information and enabling orienting shifts of attention (Babushkina and Manahan-Vaughan, 2025; Lee and Greening, 2018). During attentional tasks, the LC regulates the balance between “exploration” and “exploitation” states through the dynamic switching between tonic and phasic modes: when phasic activity is enhanced, cognitive resources are highly concentrated on the current goal, with a greater tendency toward “exploitation” of existing information to accomplish the task, leading to a marked increase in attentional stability; when tonic activity is elevated, sensitivity to other potential stimuli in the environment increases, making the individual more prone to distraction and a shift toward novel stimuli, thereby significantly enhancing attentional flexibility and a greater tendency toward “exploration” of new information (Aston-Jones and Cohen, 2005). This dual-mode regulatory mechanism enables the LC to flexibly adjust attentional states according to task demands and environmental changes, achieving a balance between stability and flexibility, which constitutes the core foundation for the efficient regulation of attention.

### 5.2 Signal-to-noise ratio enhancement in sensory processing

A core function of attention is to enhance target-related signals while suppressing background noise, enabling individuals to rapidly extract critical information from complex environments, and noradrenaline (NE) released by the LC serves as the key neurotransmitter mediating this function. By acting on *α*- and *β*-adrenergic receptors on cortical neurons, NE improves the signal-to-noise ratio (SNR) of sensory inputs at two levels: on the one hand, it suppresses spontaneous firing activity, thereby reducing background noise and minimizing interference from irrelevant information; on the other hand, it enhances sensory-evoked postsynaptic responses, boosting the neural representation strength of target-related signals and allowing critical information to dominate within neural networks (Servan-Schreiber et al., 1990; Tully and Bolshakov, 2010; Devilbiss and Waterhouse, 2000). This “signal amplification, noise suppression” effect makes target stimuli more readily captured by downstream cognitive systems and provides high-quality information input for subsequent decision-making and memory encoding (Servan-Schreiber et al., 1990; Tully and Bolshakov, 2010; Devilbiss and Waterhouse, 2000). These findings demonstrate that the LC, through NE-mediated SNR regulation, achieves the modulation of multimodal attention, providing the neurophysiological basis for attentional selection in complex information environments.

### 5.3 Modulating neural gain to influence attentional resource allocation

In addition to enhancing the SNR, the LC can also influence the distribution of attentional resources by modulating neural gain, thereby achieving an optimal allocation of cognitive resources. Neural gain reflects the slope of the neuronal response to input signals and is a key factor determining the sensitivity and efficiency of information integration (Aston-Jones and Cohen, 2005). The regulation of neural gain by NE released from the LC exhibits a characteristic inverted U-shaped profile: moderate NE levels place neural networks in a state of “efficient processing,” where neuronal sensitivity to task-relevant information is markedly enhanced, cognitive resources are highly concentrated on target information, and the efficiency of attentional resource allocation reaches its optimum. When NE levels are excessively high, neuronal sensitivity to all stimuli is markedly enhanced, and even irrelevant information receives substantial cognitive resources, leading to attentional dispersion and difficulty in focusing. When NE levels are too low, neuronal responses to input signals become sluggish, and even target-related information fails to receive adequate cognitive resources, resulting in attentional lapses and task disengagement (Aston-Jones and Cohen, 2005). This inverted U-shaped regulatory relationship has been confirmed in both sustained attention and selective attention tasks, and in decision-making and executive control tasks, LC-mediated modulation of neural gain can also promote the prioritized processing of critical information and suppress interference from irrelevant information, further enhancing overall task performance (Dix and Li, 2020; McBurney-Lin et al., 2022).

### 5.4 Reconfiguring attention-related brain networks

The LC reconfigures attention-related brain networks by modulating the functional coupling of large-scale brain networks, thereby providing network-level support for the orienting and shifting of attention. Functional neuroimaging studies have shown that when LC activity increases, functional connectivity within the ECN and the SN is significantly enhanced, whereas activity in the DMN is suppressed (Tsukahara and Engle, 2021; Hussain et al., 2023). The ECN is responsible for top-down attentional control, helping to sustain focus on goal-relevant information (Sarter et al., 2001); the SN is responsible for detecting salient or unexpected stimuli in the environment and triggering the reallocation of attentional resources (Geng et al., 2024); the DMN is associated with internally-directed thought, and suppression of its activity reduces internally-generated interference, allowing more cognitive resources to be directed toward external goal-relevant stimuli (Andrews-Hanna et al., 2014). This large-scale brain network reconfiguration facilitates a rapid shift from an internally-directed thought/mind-wandering state to a goal-oriented task-engaged state, enabling the swift reallocation of attentional resources. Moreover, LC input of thalamocortical pathways helps contributes to vigilance and supports sensory information, providing the foundation for the stable operation of sustained attention and ensuring that individuals can maintain focus on target information over extended periods (Rodenkirch et al., 2019).

In summary, through the dynamic switching between tonic and phasic firing modes, the LC achieves a balanced regulation of attentional stability and flexibility; through NE-mediated SNR optimization, it accomplishes the selective processing of target information; through the inverted U-shaped modulation of neural gain, it realizes the optimal allocation of attentional resources; and through the reconfiguration of attention-related brain networks, it provides network-level support for the orienting and shifting of attention. The precise allocation of attentional resources mediated by the LC provides the core information selection foundation for subsequent sensory evidence extraction and key information integration during cognitive decision-making, representing a critical link in the cognitive progression from “information selection” to “strategy selection.”

## 6 Locus coeruleus and decision-making

Decision-making is the core cognitive process through which an individual integrates environmental information, internal reward expectations, and behavioral goals to select a course of action, representing an advanced cognitive progression from “information selection (attention)” to “strategy selection.” High-quality decision-making not only depends heavily on the key information filtered by attention but also relies on the effective integration of sensory evidence and the rigorous evaluation of risks and benefits (Waskom and Kiani, 2018; Li et al., 2025). By precisely modulating core processes such as the exploration-exploitation balance, the efficiency of evidence accumulation, and the setting of decision thresholds, the LC serves as an important neuromodulatory node in the decision-making process, contributing to adaptive behavioral decisions (Aston-Jones and Cohen, 2005).

### 6.1 Modulation of the exploration–exploitation balance

In complex environments, individuals need to flexibly switch between “exploration” (trying new options to acquire unknown information) and “exploitation” (adhering to the current optimal strategy to obtain stable rewards), and the regulation of this balance determines the adaptiveness of decision-making. The firing patterns of the LC–NE system constitute the core neural mechanism governing this balance (Mäki-Marttunen et al., 2020). When LC tonic firing levels are high, basal NE release increases, enhancing sensitivity to novel stimuli and increasing behavioral variability, thus biasing the individual toward exploratory behavior—a state suited to gathering more cues in variable or information-poor environments. In contrast, when the LC generates prominent phasic firing, transient NE release surges, heightening focus on the current goal and enabling more efficient utilization of existing information to accomplish the task, thereby biasing the individual toward exploitative behavior—a state suited to obtaining stable rewards in stable, information-rich environments (Mäki-Marttunen et al., 2020). Through the dynamic switching between tonic and phasic firing modes, the LC flexibly adjusts the exploration–exploitation balance, providing the neurophysiological basis for dynamically optimizing decision strategies according to environmental stability, reward structure, and uncertainty.

### 6.2 Modulation of evidence accumulation efficiency

In evidence accumulation models of decision-making, such as the drift-diffusion model, decision quality is primarily determined by the speed of evidence accumulation (drift rate) and the decision threshold (Su et al., 2025). Activation of the LC–NE system enhances the responsiveness of cortical and hippocampal neurons to sensory evidence and accelerates the rate of neural information integration, thereby enabling individuals to integrate the key information selected by attention more efficiently and to improve decision accuracy without markedly increasing reaction time (Su et al., 2025). This modulatory effect is particularly pronounced under conditions of high task difficulty, in post-error trials, or in contexts of high information uncertainty, where individuals must integrate limited sensory evidence more efficiently, and phasic LC firing can transiently boost NE release, providing neuromodulatory support for evidence accumulation. Moreover, the LC–NE system not only influences low-level perceptual decisions, but also enhances the efficiency of evidence weight integration in higher-order semantic judgments and reasoning tasks, reducing cognitive biases and improving judgment accuracy, indicating that LC–NE system regulation of decision-making displays cross-level characteristics (Mækelæ and Kreis, 2024).

### 6.3 Dynamic setting of decision thresholds

The decision threshold reflects the amount of evidence an individual must accumulate before making a response and is the core factor governing the speed–accuracy trade-off. The LC–NE system modulates the decision threshold in a state-dependent manner. Through the dynamic switching between tonic and phasic firing modes, it achieves optimization of the speed–accuracy trade-off: under the tonic firing mode, elevated baseline NE levels lower the decision threshold, making individuals more likely to respond rapidly, which is suitable for time-sensitive situations with high speed demands. Under the phasic firing mode, transient burst release of NE simultaneously enhances the SNR and the drift rate of evidence accumulation, allowing individuals to improve response efficiency while accumulating sufficient evidence, thereby achieving the optimal decision of “high accuracy and high speed,” which is ideal for high-risk contexts that place a premium on accuracy (Aston-Jones and Cohen, 2005). Furthermore, in high-risk situations or when reward is highly contingent on accuracy, the LC–NE system can further elevate NE release, prompting an increase in the decision threshold, leading individuals to adopt a more cautious decision strategy to secure higher accuracy, illustrating its fine-tuned capacity for regulating decision thresholds (Dix and Li, 2020).

### 6.4 Synergistic modulation with the reward system

Decision-making is typically accompanied by reward evaluation, and the outcome of reward evaluation in turn influences decision strategies. This process involves the synergistic modulation between the LC–NE system and the midbrain DA system. The midbrain DA system encodes reward prediction errors: when actual rewards deviate from expected rewards, the DA system generates corresponding signals that adjust subsequent decision-making behavior. The LC–NE system, by contrast, amplifies the weight of key evidence by enhancing stimulus salience and information gain, enabling reward-related information to be prioritized for processing and integration (Dix and Li, 2020) The anterior cingulate cortex (ACC) plays an integrative role in this process, consolidating information on reward feedback, task conflict, and uncertainty, and in turn modulating LC activity to achieve synergistic regulation between the LC–NE and midbrain DA systems (Hassani and Tiesinga, 2024). Moreover, bidirectional projections exist between the ACC and the LC, providing the anatomical pathway substrate for this synergistic modulation. This collaborative mechanism involving multiple transmitter systems enables individuals to more accurately evaluate risks and rewards in complex reward environments, optimize value judgments and action selection, and thereby enhance the adaptiveness and effectiveness of decision-making.

In summary, through the dynamic switching between tonic and phasic firing modes, the LC achieves flexible regulation of the exploration–exploitation balance; by enhancing neuronal responsiveness to sensory evidence, it improves the efficiency of evidence accumulation; through state-dependent threshold setting, it optimizes the speed–accuracy trade-off in decision-making; and through synergistic interaction with the midbrain DA system, it refines reward-guided decision-making. The optimal decision strategy selection mediated by the LC represents a crucial step in goal-directed behavior, and the translation of decision strategies into concrete behavioral output depends on the integrative regulation of higher-level executive functions by the LC, which serves as the core strategic foundation for executive function implementation.

## 7 Locus coeruleus and executive function

Executive function is a higher-order cognitive function that integrates core capacities such as working memory, cognitive flexibility, error monitoring, and behavioral inhibition. It represents the final cognitive step from “strategy selection (decision-making)” to “behavioral execution,” and its core function is to translate the strategies formulated during decision-making into concrete, goal-directed behavior, making it essential for the performance of complex actions. The implementation of executive function relies heavily on neural network activity within the PFC (Sara and Bouret, 2012; Chen H. Y. et al., 2023). A large body of research indicates that the LC and its NE system serve as a central neuromodulatory node for executive function by modulating neural gain, network stability, and flexibility within the PFC. This regulation depends on the basal arousal state provided by vigilance and also integrates the information selection mechanisms of attention, the strategy optimization logic of decision-making, and the information storage foundation of learning and memory, thereby playing a critical role across multiple dimensions of executive function (Aston-Jones and Cohen, 2005; Rodenkirch et al., 2019; Hassani and Tiesinga, 2024).

### 7.1 Global modulation of prefrontal executive control

The PFC is the core brain region responsible for executive function. The LC sends dense noradrenergic projections to the PFC and, by acting on *α*- and *β*-adrenergic receptors on PFC neurons, modulates neural network activity within the PFC—a critical neural foundation for the PFC to exert normal executive control (Tsukahara and Engle, 2021; Hussain et al., 2023). NE released from the LC regulates the excitability, synaptic transmission efficacy, and network gain of PFC neurons. When NE input is moderate, it enhances the sustained responses of PFC neurons to task-relevant information, rendering goal representations more stable while suppressing interference from irrelevant information, thereby providing clear neural signals for the implementation of executive function (Tsukahara and Engle, 2021; Hussain et al., 2023). From a neurocomputational perspective, NE influences the stability–flexibility balance within PFC networks by modulating neural gain: on the one hand, it maintains the neural representations of current goals and task rules, ensuring behavioral stability and coherence; on the other hand, when the environment changes or task rules are adjusted, it supports strategy updating and the reconfiguration of neural representations, ensuring behavioral flexibility and adaptability. This dynamic stability–flexibility balance lies at the core of normal executive function. When NE release is moderate, PFC networks can both stably maintain task rules and flexibly respond to novel situations; however, excessively high or low NE release disrupts this balance, leading to impaired executive control (Torres et al., 2025; Jones and Graff-Radford, 2021).

### 7.2 Modulation of working memory and sustained attention

Working memory is the foundation of executive function and depends on stable, sustained neural activity within the PFC to temporarily store and manipulate task-relevant information. The LC–NE system regulates working memory by enhancing the responsiveness of PFC neurons to target-related information (Tsukahara and Engle, 2021). Appropriate NE levels strengthen the neural representation of target information within PFC networks, rendering working memory more robust, delaying the decay of attentional resources, and helping individuals sustain the storage and manipulation of key information during complex tasks. At the same time, NE enhances the PFC’s ability to suppress distracting stimuli, reducing interference from irrelevant information and ensuring that target information is processed effectively. Consistent with its regulation of other cognitive functions, the LC–NE system’s modulation of working memory also exhibits an inverted U-shaped profile: excessively high NE levels may lead to neuronal hyperexcitability and increased noise, thereby impairing the precision of working memory and rendering individuals prone to memory confusion; excessively low NE levels may result in insufficient drive and attentional lapses, making it difficult to sustain working memory and carry out complex information manipulation (Berridge and Spencer, 2016). This inverted U-shaped relationship indicates that the LC–NE system must operate within an optimal range to effectively support working memory and sustained attention, which constitutes the foundation for normal executive function.

### 7.3 Modulation of cognitive flexibility and task switching

Cognitive flexibility refers to the ability to rapidly adjust strategies, attentional allocation, or behavioral patterns in response to environmental changes or task demands, and is a key component of executive function (Jones and Graff-Radford, 2021). Its core functions are task switching and strategy updating. The LC–NE system plays a critical regulatory role in this process. When an individual needs to switch from one task rule to another, moderate activation of the LC can promote the reconfiguration of PFC networks by releasing NE to modulate the synaptic plasticity and excitability thresholds of PFC neurons, thereby reducing the “switch cost” and allowing new task rules to rapidly replace old neural representations, enabling the efficient updating of cognitive strategies (Torres et al., 2025). Simultaneously, NE can enhance the functional connectivity between the SN and the ECN, accelerating the detection of and response to changes in task rules and further improving cognitive flexibility (Torres et al., 2025). When NE levels are abnormal, cognitive flexibility is markedly impaired: excessively high NE levels render PFC networks unstable and neuronal representations difficult to sustain, leading to frequent strategy shifts and impulsive behavior, and an inability to perform tasks stably; excessively low NE levels reduce PFC network plasticity, making old neural representations difficult to suppress or update, resulting in cognitive rigidity and difficulty in task switching, and an inability to adapt to environmental changes (Ogg et al., 2025). These phenomena reflect the state-dependent nature of the LC–NE system’s regulation of executive function: only when NE levels are moderate can cognitive flexibility operate normally.

### 7.4 Role in error monitoring and behavioral adjustment

Error monitoring is a component of executive function that enables individuals to promptly detect behavioral errors or deviations between outcomes and expectations, and to rapidly adjust subsequent behavior based on feedback—a fundamental capacity for behavioral adaptation. The LC is a key neuromodulatory nucleus for error monitoring. When an error occurs or an outcome deviates from expectations, the LC typically generates phasic firing, promoting transient NE release in the PFC, which amplifies error-related signals and enhances the individual’s ability to detect errors. This process is closely associated with the enhancement of the error-related negativity (ERN), a scalp-recorded brain potential elicited upon error detection, whose amplitude is directly proportional to error monitoring sensitivity: the larger the ERN amplitude, the more sensitive the error detection. LC-mediated NE release can markedly increase ERN amplitude, thereby improving error monitoring efficiency (Ogg et al., 2025). Simultaneously, by increasing the sensitivity of PFC neurons to error-related information, NE enables individuals to timely adjust behavioral strategies and avoid repeating mistakes (Ogg et al., 2025). Thus, the LC–PFC pathway constitutes an important source of neuromodulation for error monitoring and behavioral adjustment, and the integrity of its function is closely linked to the individual’s capacity for behavioral correction.

### 7.5 Firing patterns and executive strategy selection

The dual tonic and phasic activity modes of the LC not only influence arousal, attention, and decision-making, but also affect executive strategies, thereby determining the efficiency and adaptability of executive function. Tonic activity reflects the individual’s overall level of task engagement: excessively low tonic firing leads to lack of motivation and behavioral sluggishness, significantly weakening executive function and hindering the completion of complex tasks; excessively high tonic firing tends to cause distractibility and impulsivity, disorganizing executive strategies and impairing goal-directed task performance. Phasic firing, in contrast, provides transient yet precise neuromodulation for critical events, enhances the processing of task-core information, facilitates rapid adjustments of executive strategies, and helps individuals promptly respond to unexpected situations (Ogg et al., 2025). This tonic–phasic state switching is closely linked to the “exploration–exploitation” strategy: when tonic activity is elevated, individuals are more inclined to explore new executive strategies, a state suited to contexts with ambiguous task rules; when phasic activity is enhanced, individuals tend to exploit existing effective strategies, a state appropriate for contexts with clear task rules that require stable execution (Ogg et al., 2025). Through the dynamic switching between tonic and phasic firing modes, the LC enables individuals to strike a balance between stably executing current rules and flexibly adjusting strategies, thereby optimizing the selection of executive strategies.

**Figure 2:**
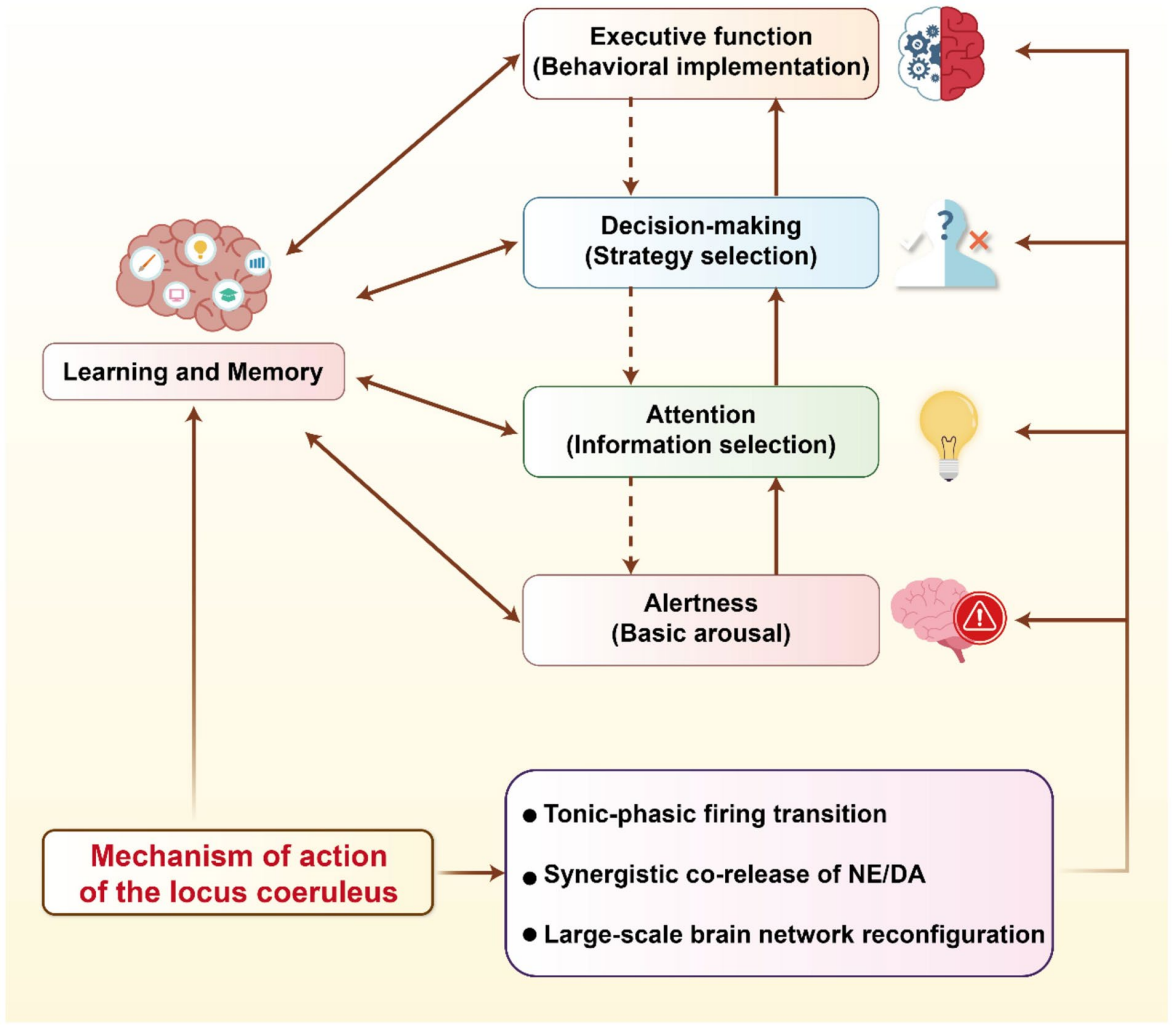
Schematic representation of the locus coeruleus (LC) as a central hub for cognitive control and learning/memory. The LC mediates cognitive function and memory processes through three core mechanisms: (1) Tonic-phasic firing transition, (2) synergistic co-release of norepinephrine (NE) and dopamine (DA), and (3) large-scale brain network reconfiguration. These mechanisms form the foundation for a hierarchical cognitive cascade, starting from basic arousal (alertness), progressing through information selection (attention), strategy selection (decision-making), and culminating in behavioral implementation (executive function). Concurrently, the LC directly underpins learning and memory, which interacts bidirectionally with all cognitive levels to enable experience-based adaptation. Executive function output feeds back to the LC, forming an adaptive regulatory loop that dynamically modulates cognitive and mnemonic processes according to environmental demands. Abbreviations used in this figure: all terms are defined in earlier sections of the main text.

In summary, the LC–NE system provides overall support for prefrontal executive control by modulating neural gain and network balance in the PFC; through inverted U-shaped modulation, it precisely regulates working memory and sustained attention; by reducing the switch cost of strategy updating, it enhances cognitive flexibility and task-switching ability; by amplifying error-related signals, it strengthens error monitoring and behavioral adjustment; and by dynamically switching between tonic and phasic firing modes, it optimizes the selection of executive strategies. The multidimensional functional regulation mediated by the LC constitutes the neural foundation for the normal execution of prefrontal executive functions, while its dysfunction serves as a critical pathological basis for executive dysfunction, marking the turning point from “normal cognitive regulation” to “pathological cognitive disorders” (Figure 2).

## 8 Discussion

This article draws on recent advances in neurophysiology, neuroscience, and clinical research to systematically review the hierarchical regulatory mechanisms of the LC in normal cognitive function, establishes the important role of the LC as a central hub for cognitive regulation in the brain, briefly analyzes the association between impaired LC regulation and pathological cognitive disorders, and constructs a theoretical framework spanning from “physiological regulation” to “pathological imbalance” The following discussion will address the core findings, innovations, pathological extensions, and current limitations and future directions of this work.

### 8.1 Core features of the LC-mediated hierarchical cognitive regulation system

The regulation of cognitive functions by the LC is not a single, isolated process but rather forms a progressively layered and mutually coordinated hierarchical system. This system is characterized by three cardinal features—integration, state-dependence, and hierarchical progression—which collectively enable the LC to efficiently modulate cognition across the whole brain. First, integration. The five core cognitive functions—learning and memory, vigilance, attention, decision-making, and executive function—are not independently modulated by the LC. Instead, their cross-functional integration is achieved through three core mechanisms: dynamic switching of firing patterns, coordinated co-release of NE and DA, and large-scale brain network reconfiguration. For instance, the switching between tonic and phasic firing modes not only regulates the level of vigilance but also influences the stability and flexibility of attention, the exploration–exploitation balance in decision-making, and the selection of executive strategies. NE-mediated optimization of the SNR provides the basis for attentional selection of information, while also supporting evidence accumulation during decision-making and the maintenance of goal representations in executive function. This integrated cross-functional regulation ensures that the various cognitive processes form an organic whole, preventing the fragmentation of cognitive operations. Second, state-dependence. The regulation of all cognitive functions by the LC follows an inverted U-shaped pattern: optimal cognitive performance is achieved only when LC activity (including firing rate and NE/DA release levels) falls within an appropriate range. Both excessively high and excessively low activity disrupt the cognitive balance, leading to a decline in cognitive efficiency. This state-dependence reflects the fine-tuned nature of LC-mediated cognitive regulation and enables individuals to dynamically adjust their cognitive state according to task demands and environmental changes. Third, hierarchical progression. The cognitive regulatory system of the LC displays a distinct characteristic of hierarchical progression, proceeding from the most fundamental level of vigilance (which serves as the prerequisite for all cognitive processes), to attention that achieves information selection, then to decision-making that completes strategy selection, and finally to executive function, which converts strategies into goal-directed actions. Each stage provides the foundation and support for the next. Meanwhile, learning and memory processes permeate the entire progression, accumulating experience that facilitates the dynamic adjustment of every stage. This enables cognitive regulation to evolve from “undifferentiated arousal” to “precise and strategic behavioral implementation.”

### 8.2 Key advances and innovations in LC-mediated regulation of cognitive functions

In recent years, advances in precise neuromodulation techniques and high-resolution imaging have yielded significant progress in understanding how the LC regulates cognitive functions, breaking through the limitations of traditional views and enriching the theoretical framework of neuromodulation in cognition. First, research on the co-release of NE and DA from the LC has overturned the long-held notion that the LC releases NE exclusively. This finding indicates that, while NE released from the LC supports rapid modulation of brain states, the co-released DA contributes to the regulation of long-term synaptic plasticity and memory consolidation, serving as a key nexus linking short-term cognitive regulation with long-term learning and memory, and considerably broadening the functional scope of LC-mediated neuromodulation. Second, studies on LC regulation at the brain-network level have elevated the investigation of cognitive modulation from single brain regions or local circuits to the level of whole-brain large-scale networks. It is now established that the LC, by modulating the functional coupling among the ECN, SN, and DMN, enables a transition from local neural activity to global cognitive states, thereby providing an entirely new perspective for understanding the mechanisms underlying whole-brain cognitive integration. Third, the adaptive gain theory has been extended, offering a unified theoretical framework for LC-mediated regulation across multiple cognitive domains. Originally formulated to account for the LC’s role in regulating the exploration–exploitation balance in attention and decision-making, recent studies have confirmed that this theory is equally applicable to other cognitive functions such as vigilance, working memory, and cognitive flexibility. This extension provides cohesive theoretical support for the hierarchical cognitive regulation system of the LC and avoids a fragmented interpretation of the regulatory mechanisms underlying different cognitive functions.

### 8.3 Disruption of normal LC regulation and shared pathological pathways in cognitive disorders

The structural and functional integrity of the LC is essential for maintaining normal cognitive regulation. As an early vulnerable node for pathological damage in the brain, the disruption of its normal regulatory mechanisms serves as a core upstream foundation for the onset and progression of pathological cognitive disorders, including cognitive dysfunction associated with neurodegenerative diseases such as AD, PD, and DLB (Ciampa et al., 2022; Sun et al., 2025; Železníková et al., 2025), as well as neurodevelopmental disorders such as ADHD and ASD (Hinshaw, 2018). Departing from the traditional approach of examining the pathological role of the LC in individual diseases, this article proposes a four-pathway common framework through which LC dysfunction gives rise to cognitive impairment. The core logic of this framework is as follows: pathological cognitive disorders fundamentally result from the disruption of the normal regulatory mechanisms of the LC; the cognitive impairments observed across different diseases are superimposed upon these four common pathways by disease-specific pathological substrates, genetic backgrounds, and target-network vulnerabilities, thereby providing a unified perspective for understanding the cross-disease common mechanisms of cognitive disorders. The four pathways are as follows.

#### 8.3.1 Pathway 1: Early LC vulnerability and pathological dissemination

The LC is intrinsically vulnerable to age-related degeneration beyond the accumulation of disease-specific proteinopathies. As one of the smallest yet most widely projecting neuromodulatory nuclei, it is exposed to high metabolic demands and oxidative stress throughout life. Its neurons are predominantly unmyelinated, which facilitates volume transmission but also renders them highly susceptible to toxins, infections, and metabolic insults. Moreover, the LC is located adjacent to the fourth ventricle and in close proximity to brain barriers with age-compromised permeability, making it an early target for blood-borne pathogens and inflammatory mediators. These factors, together with chronic stress exposure, generate a deleterious feedback loop: initial LC damage impairs noradrenergic anti-inflammatory and metabolic support, which in turn exacerbates local pathology and further weakens LC integrity, progressively driving cognitive decline with advancing age (Mather and Harley, 2016). Owing to these inherent vulnerabilities, LC neurons often exhibit structural and functional abnormalities before clinical cognitive decline becomes apparent. Furthermore, their diffuse whole-brain projections can give rise to a “hub-channel effect” for pathological propagation and network destabilization, amplifying focal pathological damage into systemic cognitive impairment (Gyoneva and Traynelis, 2013; Kang et al., 2020).

#### 8.3.2 Pathway 2: Imbalance in neuromodulator tone

The LC exhibits impaired tonic–phasic firing mode switching, leading to imbalanced NE and DA release, which disrupts the normal inverted U-shaped regulatory relationship and results in unstable whole-brain neural gain and network states, clinically manifesting as cognitive symptoms such as attentional fluctuation, cognitive fatigue, and impulsive/rigid decision-making (Young et al., 2017).

#### 8.3.3 Pathway 3: Inflammatory–phagocytic imbalance

The degeneration of LC results in reduced NE release, weakening the LC-mediated endogenous anti-inflammatory effects; microglia become excessively activated and functionally skewed, generating a chronic low-grade inflammatory microenvironment on the one hand, and aberrantly phagocytosing normal neural structures on the other, thereby exacerbating synaptic loss and functional decoupling of brain networks (Giorgi and Biagioni, 2020; Le et al., 2025).

#### 8.3.4 Pathway 4: Imbalances in synaptic plasticity and neurovascular–energy metabolism

**Figure 3:**
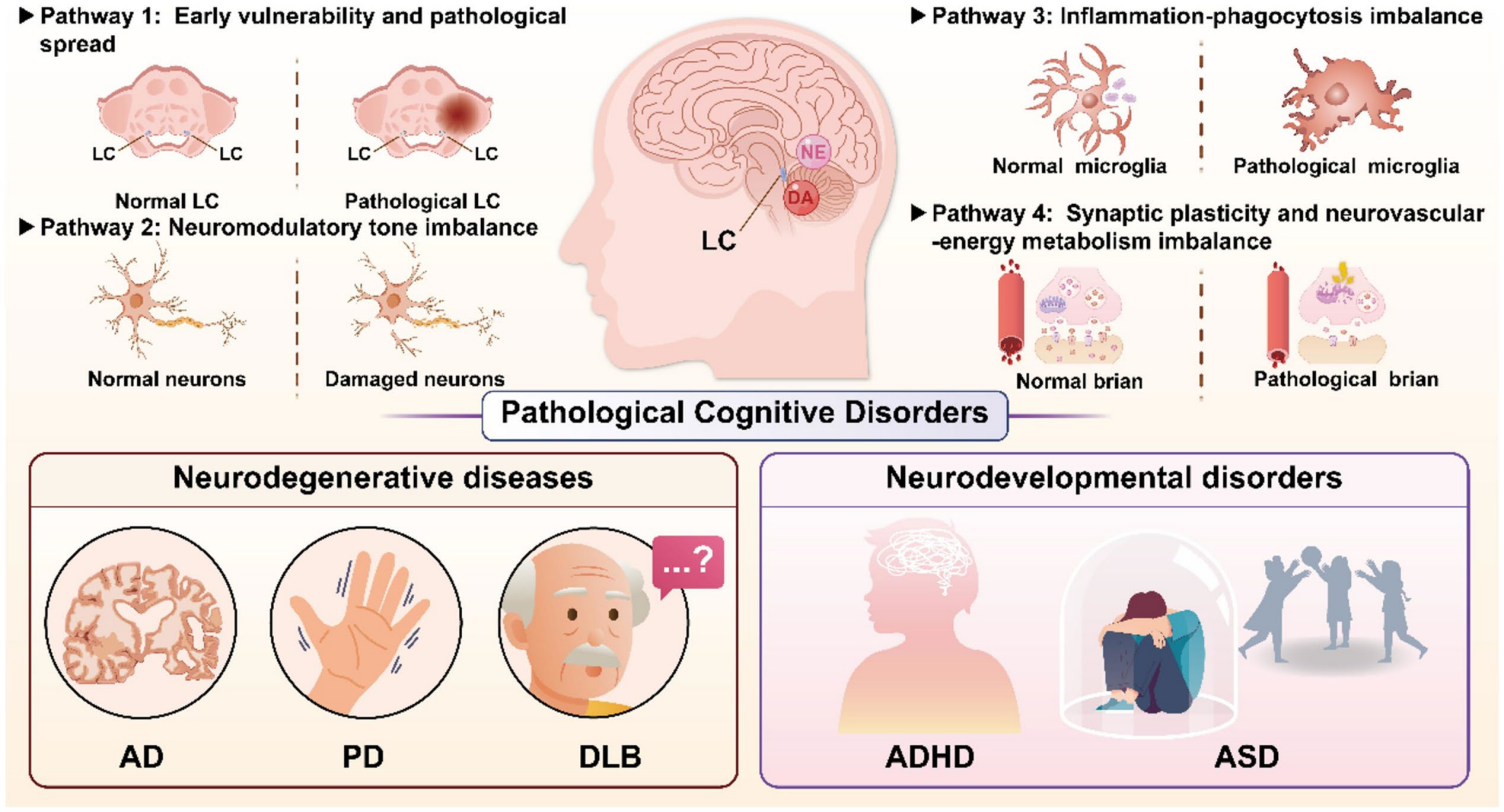
Four-pathway framework linking LC dysfunction to transdiagnostic pathological cognitive impairment. This integrative framework summarizes convergent mechanistic pathways through which structural and functional disruption of the LC contributes to cognitive dysfunction across neurodegenerative and neurodevelopmental disorders. Pathway 1 is early vulnerability and pathological spread. The LC represents an early susceptible node whose degeneration may facilitate large-scale network destabilization and propagation of pathological processes through widespread projection systems. Pathway 2 is neuromodulatory tone imbalance. Disruption of tonic–phasic firing dynamics and catecholaminergic signaling leads to impaired neural gain regulation, unstable cognitive state control, and maladaptive network reconfiguration. Pathway 3 is inflammation–phagocytosis imbalance. Reduced noradrenergic immunoregulation promotes dysregulated microglial activation, impaired clearance mechanisms, and chronic neuroinflammatory signaling that further damages neural circuits. Pathway 4 is synaptic plasticity and neurovascular–energy metabolism imbalance. LC degeneration disrupts receptor-dependent plasticity regulation, astrocyte-mediated metabolic support, and neurovascular coupling, resulting in impaired synaptic stability and reduced energetic capacity for cognitive processing. These interacting mechanisms converge to produce multidimensional cognitive impairment, manifesting across disease categories including neurodegenerative disorders (e.g., AD, PD, DLB) and neurodevelopmental disorders (e.g., ADHD and ASD). The framework emphasizes LC dysfunction as an upstream systems-level regulator of cognitive state stability, synaptic plasticity, and brain network integrity across diagnostic boundaries. Abbreviations used in this figure: all terms are defined in earlier sections of the main text.

Insufficient neuromodulator output from the LC leads to failure of its “gating regulation” of synaptic plasticity, with impaired LTP induction and excessive activation of LTD; simultaneously, neurovascular coupling mechanisms are disrupted, resulting in inadequate blood flow and energy supply to key brain regions. This creates a dual deficit in plasticity and energetic support, manifesting as unstable memory traces and working memory that is highly susceptible to interference (Gao et al., 2016; Chai et al., 2016; Sun et al., 2026). These four pathways are mutually synergistic and mutually amplifying, progressively expanding local LC dysfunction into whole-brain cognitive network destabilization, ultimately giving rise to multifaceted pathological cognitive impairment (Figure 3).

Beyond the disorders discussed above, it is noteworthy that LC–NE dysregulation has also been implicated in the cognitive impairments observed in other neuropsychiatric conditions that are frequently comorbid with dementia and age-related cognitive decline, including schizophrenia (Mäki-Marttunen et al., 2020), depressive disorder (Valdés Cabrera et al., 2026), and post-traumatic stress disorder (PTSD) (Ericson, 2026). Although these conditions were beyond the primary scope of the present review, the four-pathway framework outlined here may provide a useful lens for examining whether shared LC-related mechanisms contribute to their cognitive manifestations. Systematic investigation of this possibility represents an important direction for future research.

### 8.4 Key controversies and future directions

Despite recent progress in research on LC regulation of cognitive functions, several controversies and knowledge gaps remain, warranting further investigation. First, evidence regarding whether the human LC co-releases DA is still insufficient. While animal studies have convincingly demonstrated LC-derived DA release in the hippocampus and PFC and its role in synaptic plasticity and memory formation (Devoto et al., 2005; Takeuchi et al., 2016; Lynch, 2004; Morris, 2003; Deng et al., 2025; Kempadoo et al., 2016), direct confirmation of this mechanism in the living human brain remains lacking. Future studies combining non-human primate models with advanced human neuroimaging will be essential to clarify whether and when LC-mediated DA co-release occurs in the human brain and how it contributes to cognitive processes.

Second, *in vivo* imaging of the LC remains technically challenging because of its small size (~1–2 mm in diameter), deep pontine location, and the limited spatial resolution and SNR of conventional MRI. As discussed above, recent developments in high-resolution NM-MRI, high spatial and angular resolution diffusion MRI, high-field functional MRI, and simultaneous PET-MRI are progressively overcoming these barriers and enabling more precise in vivo quantification of LC structure, connectivity, and task-related activity (Valdés Cabrera et al., 2026; Solders et al., 2022; Sun et al., 2020). Electrophysiological approaches, combined with pupillometry, offer complementary tools for tracking LC activity with high temporal resolution (Kim and Kadlaskar, 2022). Further improvement in spatiotemporal resolution and standardization of acquisition and analysis protocols will be essential to fully characterize the role of the LC in cognitive regulation and disease. Integrating multimodal imaging with electrophysiological recordings and computational modeling will help bridge the gap between neuronal-level LC activity and systems level cognitive behavior.

Third, the synergistic regulatory mechanisms between the LC and other neuromodulator systems remain unclear. Cognitive regulation is not accomplished by the LC–NE system alone; rather, it results from the coordinated interaction of multiple neuromodulatory systems, including the dopaminergic, cholinergic, and serotonergic systems (Avery and Krichmar, 2017). Systematic dissection of these interactions and their joint contributions to cognitive state transitions represents a critical avenue for constructing a comprehensive whole-brain neuromodulatory network model.

Fourth, research on the mechanisms underlying individual differences in LC-mediated regulation of cognitive functions is lacking. Factors such as age, genetic background, and lifestyle are known to influence LC structure and function, yet how these factors shape individual variability in cognitive regulation remains poorly understood. Large-sample longitudinal cohort studies integrating multimodal LC imaging, genetic profiling, and detailed cognitive phenotyping are needed to elucidate these relationships.

Fifth, the applicability of the current four-pathway framework to other LC-associated cognitive disorders—including schizophrenia, depression, and PTSD—should be explored to determine the boundary conditions and generalizability of the proposed pathways (Ericson, 2026; Valdés Cabrera et al., 2026; Mäki-Marttunen et al., 2020).

Sixth, a critical methodological consideration concerns the interpretation of LC involvement in higher-order cognitive functions. Given its small size, widespread projections, and evolutionarily ancient origin, the LC is consistently identified in functional neuroimaging studies across a remarkably broad range of cognitive tasks. However, as Zink et al. (2021) have cogently argued, highly connected hub regions in the brain often survive statistical analyses because of their mediating role within large-scale networks, rather than because they directly or uniquely control a specific cognitive process. This carries the risk of falsely localizing higher-level cognitive functions to a single lower-level structure, when these functions may instead emerge from interactions across distributed brain areas (Eisenreich et al., 2017). The LC, with its diffuse noradrenergic projections and volume transmission mode, is particularly susceptible to this interpretive challenge: its activity may reflect the noradrenergic component of widespread network state transitions, rather than a top-down command signal for any single cognitive function. Indeed, the problem of “who controls the controller” (Zink et al., 2021; Eisenreich et al., 2017; Hazy et al., 2006) persists when the LC is presented as the apex of a cognitive hierarchy. Future studies must therefore move beyond simple structure–function associations and employ causal manipulations, computational modeling, and network-based analyses to disentangle whether the LC directly drives specific cognitive operations or primarily enables the conditions under which these operations emerge from distributed circuits. Acknowledging this lack of functional specificity does not diminish the importance of the LC in cognitive regulation; rather, it reframes its role as a critical enabling hub for whole-brain network dynamics, consistent with the hierarchical regulatory framework proposed in this review.

To address the above research gaps, future studies may proceed along four directions: first, combining non-human primate models with human brain imaging to verify the causal relationship of LC neurotransmitter co-release in the human brain; second, developing *in vivo* LC monitoring techniques with high spatiotemporal resolution and high specificity; third, systematically dissecting the interaction patterns between the LC and other neuromodulator systems to construct a whole-brain neuromodulatory network; and fourth, conducting large-sample longitudinal cohort studies to elucidate the influence of factors such as age and genetics on LC function.

## 9 Conclusion and outlook

As the primary source of noradrenergic neurons in the central nervous system, the LC is not merely an “arousal center” as traditionally conceived. Rather, by virtue of its widespread whole-brain projections, dynamic switching between tonic and phasic firing modes, coordinated co-release of NE and DA, and capacity to reconfigure large-scale brain networks, the LC serves as a central neuromodulatory hub for cognitive functions. Through three principal mechanisms—firing mode modulation, coordinated multi-transmitter modulation, and brain network reconfiguration—the LC establishes a hierarchical cognitive regulation system that progresses from basic arousal (vigilance) to information selection (attention), to strategy selection (decision-making), and ultimately to behavioral execution (executive function), with learning and memory processes running through every link of this system to provide experiential support for the dynamic adjustment of each cognitive stage. This system is characterized by three cardinal features—integration, state-dependence, and hierarchical progression—with each cognitive stage building upon and synergizing with the others, forming a complete neuromodulatory network for cognitive processing in the brain and supporting the efficient and flexible coordination of cognitive functions.

The LC occupies a pivotal position in both normal cognitive processing and pathological cognitive disorders. In-depth investigation of its hierarchical regulatory mechanisms can not only deepen our understanding of the principles underlying brain cognition but also provide key theoretical support and translational pathways for the early diagnosis, precise subtyping, and targeted treatment of cognitive disorders. With continued advances in neuroimaging, neuromodulation, and cross-species research techniques, the LC is poised to demonstrate significant scientific value and translational potential in the fields of cognitive neuroscience and clinical neurology.

## References

[ref1] Anderson M. C. Bunce J. G. Barbas H. (2016). Prefrontal-hippocampal pathways underlying inhibitory control over memory. Neurobiol. Learn. Mem. 134, 145–161. doi: 10.1016/j.nlm.2015.11.008, 26642918 PMC5106245

[ref2] Andrews-Hanna J. R. Smallwood J. Spreng R. N. (2014). The default network and self-generated thought: component processes, dynamic control, and clinical relevance. Ann. N. Y. Acad. Sci. 1316, 29–52. doi: 10.1111/nyas.12360, 24502540 PMC4039623

[ref3] Arnsten A. F. (2011). Catecholamine influences on dorsolateral prefrontal cortical networks. Biol. Psychiatry 69, e89–e99. doi: 10.1016/j.biopsych.2011.01.027, 21489408 PMC3145207

[ref4] Aston-Jones G. Bloom F. E. (1981). Norepinephrine-containing locus coeruleus neurons in behaving rats exhibit pronounced responses to non-noxious environmental stimuli. J. Neurosci. 1, 887–900. doi: 10.1523/JNEUROSCI.01-08-00887.1981, 7346593 PMC6564231

[ref5] Aston-Jones G. Cohen J. D. (2005). An integrative theory of locus coeruleus-norepinephrine function: adaptive gain and optimal performance. Annu. Rev. Neurosci. 28, 403–450. doi: 10.1146/annurev.neuro.28.061604.135709, 16022602

[ref6] Avery M. C. Krichmar J. L. (2017). Neuromodulatory systems and their interactions: a review of models, theories, and experiments. Front. Neural Circuits 11:108. doi: 10.3389/fncir.2017.00108, 29311844 PMC5744617

[ref7] Babushkina N. Manahan-Vaughan D. (2022). Frequency-dependency of the involvement of dopamine D1/D5 and beta-adrenergic receptors in hippocampal LTD triggered by locus coeruleus stimulation. Hippocampus 32, 449–465. doi: 10.1002/hipo.23419, 35478421

[ref8] Babushkina N. Manahan-Vaughan D. (2025). The modulation by the locus Coeruleus of recent and remote memory retrieval is activity-dependent. Hippocampus 35:e70004. doi: 10.1002/hipo.70004, 39980081 PMC11842585

[ref9] Berridge C. W. Spencer R. C. (2016). Differential cognitive actions of norepinephrine a2 and a1 receptor signaling in the prefrontal cortex. Brain Res. 1641, 189–196. doi: 10.1016/j.brainres.2015.11.024, 26592951 PMC4876052

[ref10] Burgoyne A. P. Engle R. W. (2020). Attention control: a cornerstone of higher-order cognition. Curr. Dir. Psychol. Sci. 1–7.

[ref11] Chai G. S. Wang Y. Y. Yasheng A. Zhao P. (2016). Beta 2-adrenergic receptor activation enhances neurogenesis in Alzheimer's disease mice. Neural Regen. Res. 11, 1617–1624. doi: 10.4103/1673-5374.193241, 27904493 PMC5116841

[ref12] Chen Z. Jiang T. Yin X. Li B. Tan Z. Guo J. (2023). The increased functional connectivity between the locus coeruleus and supramarginal gyrus in insomnia disorder with acupuncture modulation. Front. Neurosci. 17:1131916. doi: 10.3389/fnins.2023.1131916, 37152608 PMC10157050

[ref13] Chen H. Y. Parent J. H. Ciampa C. J. Dahl M. J. Hämmerer D. Maass A. . (2023). Interactive effects of locus coeruleus structure and catecholamine synthesis capacity on cognitive function. Front. Aging Neurosci. 15:1236335. doi: 10.3389/fnagi.2023.1236335, 37744395 PMC10516288

[ref14] Ciampa C. J. Parent J. H. Harrison T. M. Fain R. M. Betts M. J. Maass A. . (2022). Associations among locus coeruleus catecholamines, tau pathology, and memory in aging. Neuropsychopharmacology 47, 1106–1113. doi: 10.1038/s41386-022-01269-6, 35034099 PMC8938463

[ref15] Clewett D. Huang R. Davachi L. (2025). Locus coeruleus activation "resets" hippocampal event representations and separates adjacent memories. Neuron 113, 2521–2535.e8. doi: 10.1016/j.neuron.2025.05.013, 40482639

[ref16] Dahl M. J. Mather M. Werkle-Bergner M. Kennedy B. L. Guzman S. Hurth K. . (2022). Locus coeruleus integrity is related to tau burden and memory loss in autosomal-dominant Alzheimer's disease. Neurobiol. Aging 112, 39–54. doi: 10.1016/j.neurobiolaging.2021.11.006, 35045380 PMC8976827

[ref17] Deng Z. Fei X. Zhang S. Xu M. (2025). A time window for memory consolidation during NREM sleep revealed by cAMP oscillation. Neuron 113, 1983–1997.e7. doi: 10.1016/j.neuron.2025.03.020, 40233747

[ref18] Devilbiss D. M. Waterhouse B. D. (2000). Norepinephrine exhibits two distinct profiles of action on sensory cortical neuron responses to excitatory synaptic stimuli. Synapse 37, 273–282. doi: 10.1002/1098-2396(20000915)37:4<273::AID-SYN4>3.0.CO;2-#, 10891864

[ref19] Devoto P. Flore G. Saba P. Fà M. Gessa G. L. (2005). Co-release of noradrenaline and dopamine in the cerebral cortex elicited by single train and repeated train stimulation of the locus coeruleus. BMC Neurosci. 6:31. doi: 10.1186/1471-2202-6-31, 15865626 PMC1134661

[ref20] Dix A. Li S. C. (2020). Incentive motivation improves numerosity discrimination: insights from pupillometry combined with drift-diffusion modelling. Sci. Rep. 10:2608. doi: 10.1038/s41598-020-59415-3, 32054923 PMC7018719

[ref21] Eisenreich B. R. Akaishi R. Hayden B. Y. (2017). Control without controllers: toward a distributed neuroscience of executive control. J. Cogn. Neurosci. 29, 1684–1698. doi: 10.1162/jocn_a_01139, 28430042 PMC7162733

[ref22] Ericson M. (2026). Stress-related cognitive dysfunction as a problem of neuromodulatory regulation. Neurosci. Biobehav. Rev. 187:106757. doi: 10.1016/j.neubiorev.2026.106757, 42142685

[ref23] Fukabori R. Iguchi Y. (2020). Enhanced retrieval of taste associative memory by Chemogenetic activation of locus Coeruleus norepinephrine neurons. J. Neurosci. 40, 8367–8385. doi: 10.1523/JNEUROSCI.1720-20.2020, 32994339 PMC7577602

[ref24] Galgani A. Giorgi F. S. (2023). Exploring the role of locus Coeruleus in Alzheimer's disease: a comprehensive update on MRI studies and implications. Curr. Neurol. Neurosci. Rep. 23, 925–936. doi: 10.1007/s11910-023-01324-9, 38064152 PMC10724305

[ref25] Gálvez-Márquez D. K. Urrego-Morales O. Rodríguez-Durán L. F. Bermudez-Rattoni F. (2025). Photostimulation of locus coeruleus CA1 catecholaminergic terminals reversed spatial memory impairment in an Alzheimer's disease mouse model. Psychopharmacology 243, 831–850. doi: 10.1007/s00213-025-06885-w 40924167 PMC13035695

[ref26] Gao V. Suzuki A. Magistretti P. J. Lengacher S. Pollonini G. Steinman M. Q. . (2016). Astrocytic β2-adrenergic receptors mediate hippocampal long-term memory consolidation. Proc. Natl. Acad. Sci. USA 113, 8526–8531. doi: 10.1073/pnas.1605063113, 27402767 PMC4968707

[ref27] Geng H. Xu P. Aleman A. Qin S. Luo Y. J. (2024). Dynamic Organization of Large-scale Functional Brain Networks Supports Interactions between Emotion and Executive Control. Neurosci. Bull. 40, 981–991. doi: 10.1007/s12264-023-01168-w, 38261252 PMC11250766

[ref28] Giorgi F. S. Biagioni F. (2020). Locus coeruleus modulates neuroinflammation in Parkinsonism and dementia. Int. J. Mol. Sci. 21:8630. doi: 10.3390/ijms21228630, 33207731 PMC7697920

[ref29] Gyoneva S. Traynelis S. F. (2013). Norepinephrine modulates the motility of resting and activated microglia via different adrenergic receptors. J. Biol. Chem. 288, 15291–15302. doi: 10.1074/jbc.M113.458901, 23548902 PMC3663549

[ref30] Hassani S. A. Tiesinga P. (2024). Noradrenergic alpha-2a receptor stimulation enhances prediction error signaling and updating of attention sets in anterior cingulate cortex and striatum. Nat. Commun. 15:9905. doi: 10.1038/s41467-024-54395-8 39548091 PMC11568163

[ref31] Hazy T. E. Frank M. J. O'reilly R. C. (2006). Banishing the homunculus: making working memory work. Neuroscience 139, 105–118. doi: 10.1016/j.neuroscience.2005.04.067, 16343792

[ref32] Hinshaw S. P. (2018). Attention deficit hyperactivity disorder (ADHD): controversy, developmental mechanisms, and multiple levels of analysis. Annu. Rev. Clin. Psychol. 14, 291–316. doi: 10.1146/annurev-clinpsy-050817-084917, 29220204

[ref33] Huo C. Lombardi F. (2024). Role of the locus coeruleus arousal promoting neurons in maintaining brain criticality across the sleep-wake cycle. J. Neurosci. 44:e1939232024. doi: 10.1523/jneurosci.1939-23.2024, 38951035 PMC11358608

[ref34] Hussain S. Menchaca I. Shalchy M. A. Yaghoubi K. Langley J. Seitz A. R. . (2023). Locus coeruleus integrity predicts ease of attaining and maintaining neural states of high attentiveness. Brain Res. Bull. 202:110733. doi: 10.1016/j.brainresbull.2023.110733, 37586427

[ref35] James T. Kula B. Choi S. Khan S. S. Bekar L. K. Smith N. A. (2021). Locus coeruleus in memory formation and Alzheimer's disease. Eur. J. Neurosci. 54, 6948–6959. doi: 10.1111/ejn.15045, 33190318 PMC8121900

[ref36] Jones D. T. Graff-Radford J. (2021). Executive dysfunction and the prefrontal cortex. Continuum 27, 1586–1601. doi: 10.1212/con.0000000000001009, 34881727

[ref37] Kang S. S. Liu X. Ahn E. H. Xiang J. Manfredsson F. P. Yang X. . (2020). Norepinephrine metabolite DOPEGAL activates AEP and pathological tau aggregation in locus coeruleus. J. Clin. Invest. 130, 422–437. doi: 10.1172/JCI130513, 31793911 PMC6934194

[ref38] Kempadoo K. A. Mosharov E. V. Choi S. J. Sulzer D. Kandel E. R. (2016). Dopamine release from the locus coeruleus to the dorsal hippocampus promotes spatial learning and memory. Proc. Natl. Acad. Sci. USA 113, 14835–14840. doi: 10.1073/pnas.1616515114, 27930324 PMC5187750

[ref39] Kim Y. Kadlaskar G. (2022). Measures of tonic and phasic activity of the locus coeruleus-norepinephrine system in children with autism spectrum disorder: an event-related potential and pupillometry study. Autism Res. 15, 2250–2264. doi: 10.1002/aur.2820, 36164264 PMC9722557

[ref40] Kim Y. Schneider D. W. Keehn B. (2026). An investigation of attentional networks, the locus coeruleus—norepinephrine system, and autism and ADHD traits. J. Atten. Disord. 30, 82–98. doi: 10.1177/10870547251365031 40932445

[ref41] Langley J. Hussain S. (2022). Impact of locus Coeruleus and its projections on memory and aging. Brain Connect. 12, 223–233. doi: 10.1089/brain.2020.0947, 34139886 PMC9058864

[ref42] Le L. H. D. Feidler A. M. Rodriguez L. C. Cealie M. Plunk E. Li H. . (2025). Noradrenergic signaling controls Alzheimer's disease pathology via activation of microglial β2 adrenergic receptors. Brain Behav. Immun. 128, 307–322. doi: 10.1016/j.bbi.2025.04.022, 40245958 PMC12286739

[ref43] Lee T. H. Greening S. G. (2018). Arousal increases neural gain via the locus coeruleus-norepinephrine system in younger adults but not in older adults. Nat. Hum. Behav. 2, 356–366. doi: 10.1038/s41562-018-0344-1, 30320223 PMC6176734

[ref44] Lefton K. B. Wu Y. (2025). Norepinephrine signals through astrocytes to modulate synapses. Science 388, 776–783. doi: 10.1126/science.adq5480, 40373122 PMC12309572

[ref45] Li H. H. Sprague T. C. Yoo A. H. (2025). Neural mechanisms of resource allocation in working memory. Sci. Adv. 11:eadr8015. doi: 10.1126/sciadv.adr8015, 40203109 PMC11980857

[ref46] Liebe T. Kaufmann J. Hämmerer D. Betts M. Walter M. (2022). In vivo tractography of human locus coeruleus-relation to 7T resting state fMRI, psychological measures and single subject validity. Mol. Psychiatry 27, 4984–4993. doi: 10.1038/s41380-022-01761-x, 36117208 PMC9763100

[ref47] Loughlin S. E. Foote S. L. Bloom F. E. (1986). Efferent projections of nucleus locus coeruleus: topographic organization of cells of origin demonstrated by three-dimensional reconstruction. Neuroscience 18, 291–306. doi: 10.1016/0306-4522(86)90155-7, 3736860

[ref48] Luskin A. T. Li L. (2025). Heterogeneous pericoerulear neurons tune arousal and exploratory behaviours. Nature 643, 437–447. doi: 10.1038/s41586-025-08952-w, 40335695 PMC12240712

[ref49] Lynch M. A. (2004). Long-term potentiation and memory. Physiol. Rev. 84, 87–136. doi: 10.1152/physrev.00014.2003, 14715912

[ref50] Mækelæ M. J. Kreis I. V. (2024). Teleological reasoning bias is predicted by pupil dynamics: evidence for the extensive integration account of bias in reasoning. Psychophysiology 61:e14532. doi: 10.1111/psyp.14532, 38282116

[ref51] Mahoney H. L. Schmidt T. M. (2024). The cognitive impact of light: illuminating ipRGC circuit mechanisms. Nat. Rev. Neurosci. 25, 159–175. doi: 10.1038/s41583-023-00788-5, 38279030

[ref52] Mäki-Marttunen V. Andreassen O. A. Espeseth T. (2020). The role of norepinephrine in the pathophysiology of schizophrenia. Neurosci. Biobehav. Rev. 118, 298–314. doi: 10.1016/j.neubiorev.2020.07.038, 32768486

[ref53] Matchett B. J. Grinberg L. T. (2021). The mechanistic link between selective vulnerability of the locus coeruleus and neurodegeneration in Alzheimer's disease. Acta Neuropathol. Commun. 141, 631–650. doi: 10.1007/s00401-020-02248-1, 33427939 PMC8043919

[ref54] Mather M. Clewett D. Sakaki M. Harley C. W. (2016). Norepinephrine ignites local hotspots of neuronal excitation: how arousal amplifies selectivity in perception and memory. Behav. Brain Sci. 39:e200. doi: 10.1017/S0140525X15000667, 26126507 PMC5830137

[ref55] Mather M. Harley C. W. (2016). The locus coeruleus: essential for maintaining cognitive function and the aging brain. Trends Cogn. Sci. 20, 214–226. doi: 10.1016/j.tics.2016.01.001, 26895736 PMC4761411

[ref56] McBurney-Lin J. Vargova G. Garad M. Zagha E. Yang H. (2022). The locus coeruleus mediates behavioral flexibility. Cell Rep. 41:111534. doi: 10.1016/j.celrep.2022.111534, 36288712 PMC9662304

[ref57] Moncada D. (2017). Evidence of VTA and LC control of protein synthesis required for the behavioral tagging process. Neurobiol. Learn. Mem. 138, 226–237. doi: 10.1016/j.nlm.2016.06.003, 27291857

[ref58] Morris R. G. (2003). Long-term potentiation and memory. Philos. Trans. R. Soc. Lond. Ser. B Biol. Sci. 358, 643–647. doi: 10.1098/rstb.2002.1230, 12740109 PMC1693171

[ref59] Novitskaya Y. Sara S. J. Logothetis N. K. Eschenko O. (2016). Ripple-triggered stimulation of the locus coeruleus during post-learning sleep disrupts ripple/spindle coupling and impairs memory consolidation. Learn. Mem. 23, 238–248. doi: 10.1101/lm.040923.115, 27084931 PMC4836638

[ref60] O'dell T. J. Connor S. A. Guglietta R. Nguyen P. V. (2015). β-Adrenergic receptor signaling and modulation of long-term potentiation in the mammalian hippocampus. Learn. Mem. 22, 461–471. doi: 10.1101/lm.031088.113, 26286656 PMC4561407

[ref61] Ogg M. C. Lansdell B. J. Franks H. T. Hughes A. C. Lee J. Nolen H. G. . (2025). Locus coeruleus norepinephrine neurons facilitate orbitofrontal cortex remapping and behavioral flexibility. Cell Rep. 44:116687. doi: 10.1016/j.celrep.2025.116687, 41389209

[ref62] Oken B. S. Salinsky M. C. Elsas S. M. (2006). Vigilance, alertness, or sustained attention: physiological basis and measurement. Clin. Neurophysiol. 117, 1885–1901. doi: 10.1016/j.clinph.2006.01.017, 16581292 PMC2865224

[ref63] Otmakhova N. A. Lisman J. E. (1998). D1/D5 dopamine receptors inhibit depotentiation at CA1 synapses via cAMP-dependent mechanism. J. Neurosci. 18, 1270–1279. doi: 10.1523/JNEUROSCI.18-04-01270.1998, 9454837 PMC6792711

[ref64] Rivera-Maya O. B. Ortiz-Robles C. D. Palacios-Valladares J. R. Calderón-Aranda E. S. (2024). Dopamine D1-like receptor stimulation induces CREB, arc, and BDNF dynamic changes in differentiated SH-SY5Y cells. Neurochem. Res. 50:35. doi: 10.1007/s11064-024-04293-8, 39601897 PMC11602804

[ref65] Rodenkirch C. Liu Y. Schriver B. J. Wang Q. (2019). Locus coeruleus activation enhances thalamic feature selectivity via norepinephrine regulation of intrathalamic circuit dynamics. Nat. Neurosci. 22, 120–133. doi: 10.1038/s41593-018-0283-1, 30559472 PMC6301066

[ref66] Sales A. C. Friston K. J. (2019). Locus coeruleus tracking of prediction errors optimises cognitive flexibility: an active inference model. PLoS Comput. Biol. 15:e1006267. doi: 10.1371/journal.pcbi.1006267, 30608922 PMC6334975

[ref67] Sara S. J. (2009). The locus coeruleus and noradrenergic modulation of cognition. Nat. Rev. Neurosci. 10, 211–223. doi: 10.1038/nrn2573, 19190638

[ref68] Sara S. J. Bouret S. (2012). Orienting and reorienting: the locus coeruleus mediates cognition through arousal. Neuron 76, 130–141. doi: 10.1016/j.neuron.2012.09.011, 23040811

[ref69] Sarter M. Givens B. Bruno J. P. (2001). The cognitive neuroscience of sustained attention: where top-down meets bottom-up. Brain Res. Brain Res. Rev. 35, 146–160. doi: 10.1016/S0165-0173(01)00044-3, 11336780

[ref70] Schwarz L. A. Miyamichi K. Gao X. J. Beier K. T. Weissbourd B. DeLoach K. E. . (2015). Viral-genetic tracing of the input-output organization of a central noradrenaline circuit. Nature 524, 88–92. doi: 10.1038/nature14600, 26131933 PMC4587569

[ref71] Servan-Schreiber D. Printz H. Cohen J. D. (1990). A network model of catecholamine effects: gain, signal-to-noise ratio, and behavior. Science 249, 892–895. doi: 10.1126/science.2392679, 2392679

[ref72] Solders S. K. Galinsky V. L. Clark A. L. Sorg S. F. Weigand A. J. Bondi M. W. . (2022). Diffusion MRI tractography of the locus coeruleus-transentorhinal cortex connections using GO-ESP. Magn. Reson. Med. 87, 1816–1831. doi: 10.1002/mrm.29088, 34792198 PMC8810611

[ref73] Su S. Vanvoorden T. Le Denmat P. Zénon A. Braconnier C. Duque J. (2025). Transcutaneous vagus nerve stimulation boosts accuracy during perceptual decision-making. Brain Stimul. 18, 975–986. doi: 10.1016/j.brs.2025.04.020, 40311845

[ref74] Sun S. Madge V. Djordjevic J. Gagnon J. F. Collins D. L. Dagher A. . (2025). Selective effects of substantia Nigra and locus Coeruleus degeneration on cognition in Parkinson's disease. Mov. Disord. 40, 844–854. doi: 10.1002/mds.30148, 39945211 PMC12089896

[ref75] Sun L. Peng X. L. Zi H. X. Zhang Z.-K. Gong Y.-C. Li J. . (2026). Globally patterned locus coeruleus-norepinephrine neuron-pericyte coupling orchestrates brain-wide vascular dynamics. Neuron 114, 287–306.e9. doi: 10.1016/j.neuron.2025.10.010, 41232533

[ref76] Sun W. Tang Y. Qiao Y. Ge X. Mather M. Ringman J. M. . (2020). A probabilistic atlas of locus coeruleus pathways to transentorhinal cortex for connectome imaging in Alzheimer's disease. NeuroImage 223:117301. doi: 10.1016/j.neuroimage.2020.117301, 32861791 PMC7797167

[ref77] Swanson L. W. (1976). The locus coeruleus: a cytoarchitectonic, Golgi and immunohistochemical study in the albino rat. Brain Res. 110, 39–56. doi: 10.1016/0006-8993(76)90207-9, 776360

[ref78] Takeuchi T. Duszkiewicz A. J. Sonneborn A. Spooner P. A. Yamasaki M. Watanabe M. . (2016). Locus coeruleus and dopaminergic consolidation of everyday memory. Nature 537, 357–362. doi: 10.1038/nature19325, 27602521 PMC5161591

[ref79] Torres A. S. Robison M. K. Brewer G. A. (2025). The role of the LC-NE system in attention: from cells, to systems, to sensory-motor control. Neurosci. Biobehav. Rev. 175:106233. doi: 10.1016/j.neubiorev.2025.106233, 40412462

[ref80] Toyoda H. Won J. Kim W. Kim H. Davy O. Saito M. . (2022). The nature of noradrenergic volume transmission from locus Coeruleus to brainstem mesencephalic trigeminal sensory neurons. Front. Cell. Neurosci. 16:841239. doi: 10.3389/fncel.2022.841239, 35558874 PMC9087804

[ref81] Trujillo P. Aumann M. A. Claassen D. O. (2024). Neuromelanin-sensitive MRI as a promising biomarker of catecholamine function. Brain 147, 337–351. doi: 10.1093/brain/awad300, 37669320 PMC10834262

[ref82] Tsukahara J. S. Engle R. W. (2021). Fluid intelligence and the locus coeruleus-norepinephrine system. Proc. Natl. Acad. Sci. USA 118:e2110630118. doi: 10.1073/pnas.2110630118, 34764223 PMC8609644

[ref83] Tully K. Bolshakov V. Y. (2010). Emotional enhancement of memory: how norepinephrine enables synaptic plasticity. Mol. Brain 3:15. doi: 10.1186/1756-6606-3-15, 20465834 PMC2877027

[ref84] Unsworth N. Robison M. K. (2017). A locus coeruleus-norepinephrine account of individual differences in working memory capacity and attention control. Psychon. Bull. Rev. 24, 1282–1311. doi: 10.3758/s13423-016-1220-5, 28108977

[ref85] Valdés Cabrera D. Calarco N. Cassidy C. M. Voineskos A. Diniz B. S. Nikolova Y. S. (2026). Locus coeruleus microstructure and connectivity as novel markers of depression and cognitive dysfunction in older adults. Biol. Psychiatry Cogn. Neurosci. Neuroimaging 11, 366–377. doi: 10.1016/j.bpsc.2025.10.007, 41110552

[ref86] Waskom M. L. Kiani R. (2018). Decision making through integration of sensory evidence at prolonged timescales. Curr. Biol. 28, 3850–3856.e9. doi: 10.1016/j.cub.2018.10.021, 30471996 PMC6279571

[ref87] Young C. B. Raz G. Everaerd D. Beckmann C. F. Tendolkar I. Hendler T. . (2017). Dynamic shifts in large-scale brain network balance as a function of arousal. J. Neurosci. 37, 281–290. doi: 10.1523/JNEUROSCI.1759-16.2016, 28077708 PMC6596574

[ref88] Železníková Ž. Nováková L. Vojtíšek L. Brabenec L. Mitterová K. Morávková I. . (2025). Early changes in the locus coeruleus in mild cognitive impairment with Lewy bodies. Mov. Disord. 40, 276–284. doi: 10.1002/mds.30058, 39535454 PMC11832806

[ref89] Zhou W. Chu H. Y. (2025). Progressive noradrenergic degeneration and motor cortical dysfunction in Parkinson's disease. Acta Pharmacol. Sin. 46, 829–835. doi: 10.1038/s41401-024-01428-z, 39592731 PMC11950218

[ref90] Zink N. Lenartowicz A. Markett S. (2021). A new era for executive function research: on the transition from centralized to distributed executive functioning. Neurosci. Biobehav. Rev. 124, 235–244. doi: 10.1016/j.neubiorev.2021.02.011, 33582233 PMC8420078

